# Supplementary material for: A global meta-analysis on the drivers of salt marsh planting success and implications for ecosystem services
Source: Nat Commun. 2024 Apr 29;15:3643. doi: 10.1038/s41467-024-47769-5 (PMC11059165; doi:10.1038/s41467-024-47769-5)
Supplement: Supplementary file 1 — Supplementary Information [file 41467_2024_47769_MOESM1_ESM.pdf]

Supplementary Information for

A global meta-analysis on the drivers of salt marsh planting success and implications for ecosystem services

Authors list

Zezheng Liu[1], Sergio Fagherazzi[2], Qiang He[3], Olivier Gourgue[4], Junhong Bai[1,5], Xinhui Liu[1,6], Chiyuan Miao[7], Zhan Hu[8,9,10*], Baoshan Cui[1,5,6]*

Affiliations

[1]State Key Laboratory of Water Environmental Simulation, School of Environment, Beijing Normal University, Beijing 100875, China

[2]Department of Earth and Environment, Boston University, Massachusetts 02215, USA

[3]Coastal Ecology Lab, MOE Key Laboratory for Biodiversity Science and Ecological Engineering, School of Life Sciences, Fudan University, Shanghai 200438, China

[4]Operational Directorate Natural Environment, Royal Belgian Institute of Natural Sciences, 1000 Brussels, Belgium

[5]Yellow River Estuary Wetland Ecosystem Observation and Research Station, Ministry of Education, Shandong 257500, China

[6]Research and Development Center for Watershed Environmental Eco-Engineering, Beijing Normal University at Zhuhai, Zhuhai 519087, China

[7]State Key Laboratory of Earth Surface Processes and Resource Ecology, Faculty of Geographical Science, Beijing Normal University, Beijing 100875, China

[8]School of Marine Sciences, Sun Yat-Sen University, and Southern Marine Science and Engineering Guangdong Laboratory (Zhuhai), Zhuhai 519000, China

[9]Guangdong Provincial Key Laboratory of Marine Resources and Coastal Engineering, Guangzhou 510000, China

[10]Pearl River Estuary Marine Ecosystem Research Station, Ministry of Education, Zhuhai 519000, China

*Correspondence to: Baoshan Cui, cuibs@bnu.edu.cn; Zhan Hu, huzh9@mail.sysu.edu.cn

Supplementary Figures

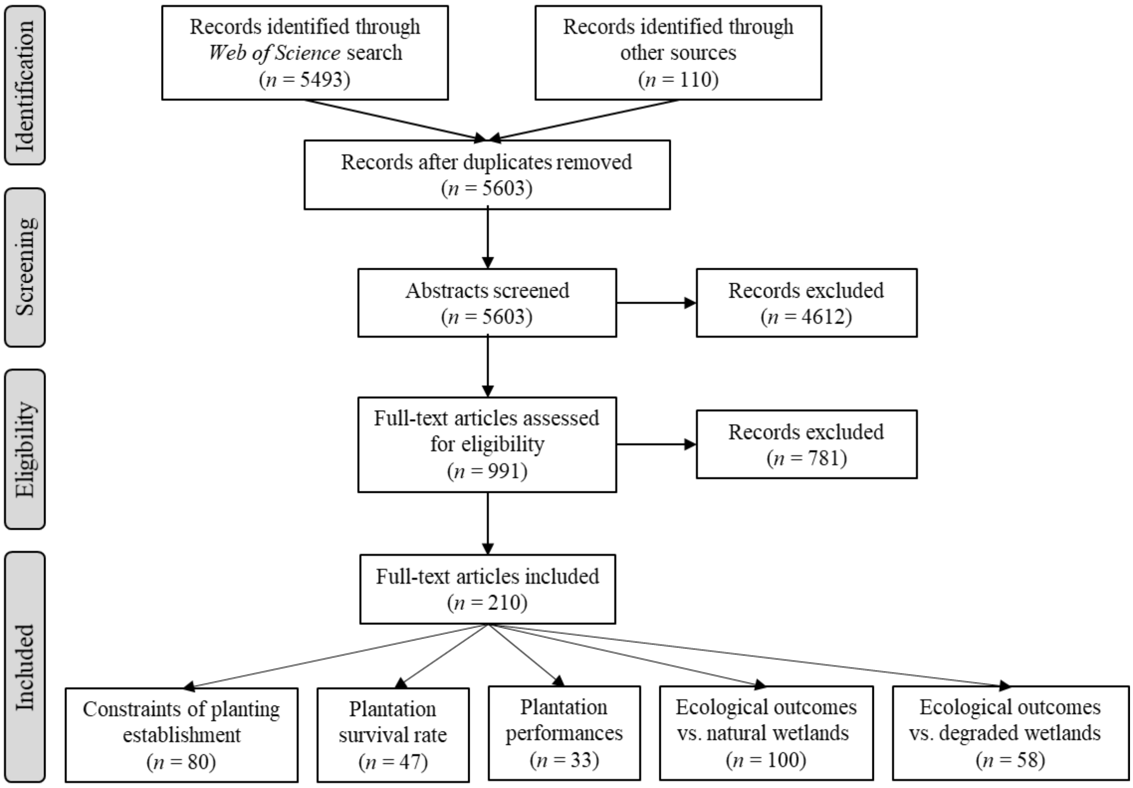

Supplementary Figure 1. PRISMA flow diagram showing the procedure used to select the publications.

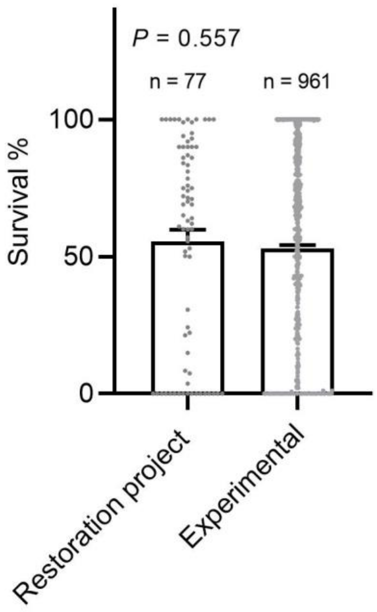

Supplementary Figure 2. Differences in survival rate (± SE) between restoration projects and experimental manipulations of plantings. Differences in survival rate were assessed using two-tailed Student t-tests. Source data are provided as a Source Data file.

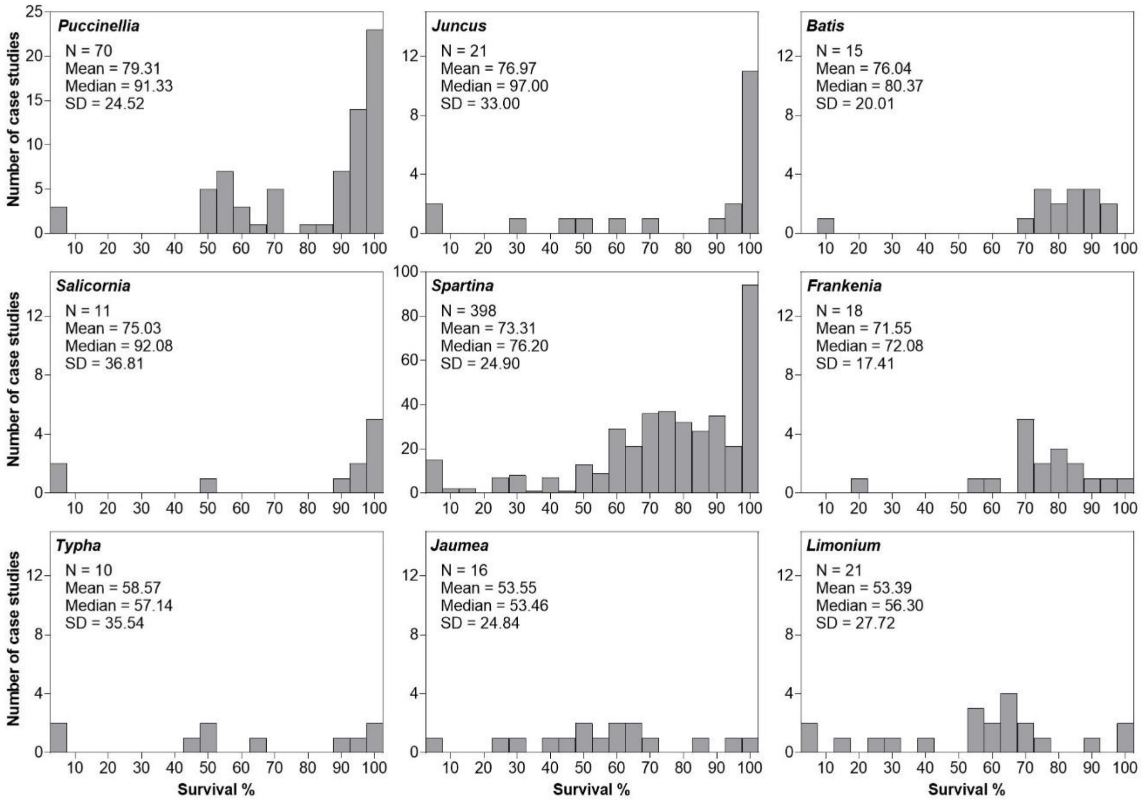

Supplementary Figure 3. Frequency distribution of survival of species with survival rates higher than the mean value (52.8%). Median values and observations are indicated by numbers. Source data are provided as a Source Data file.

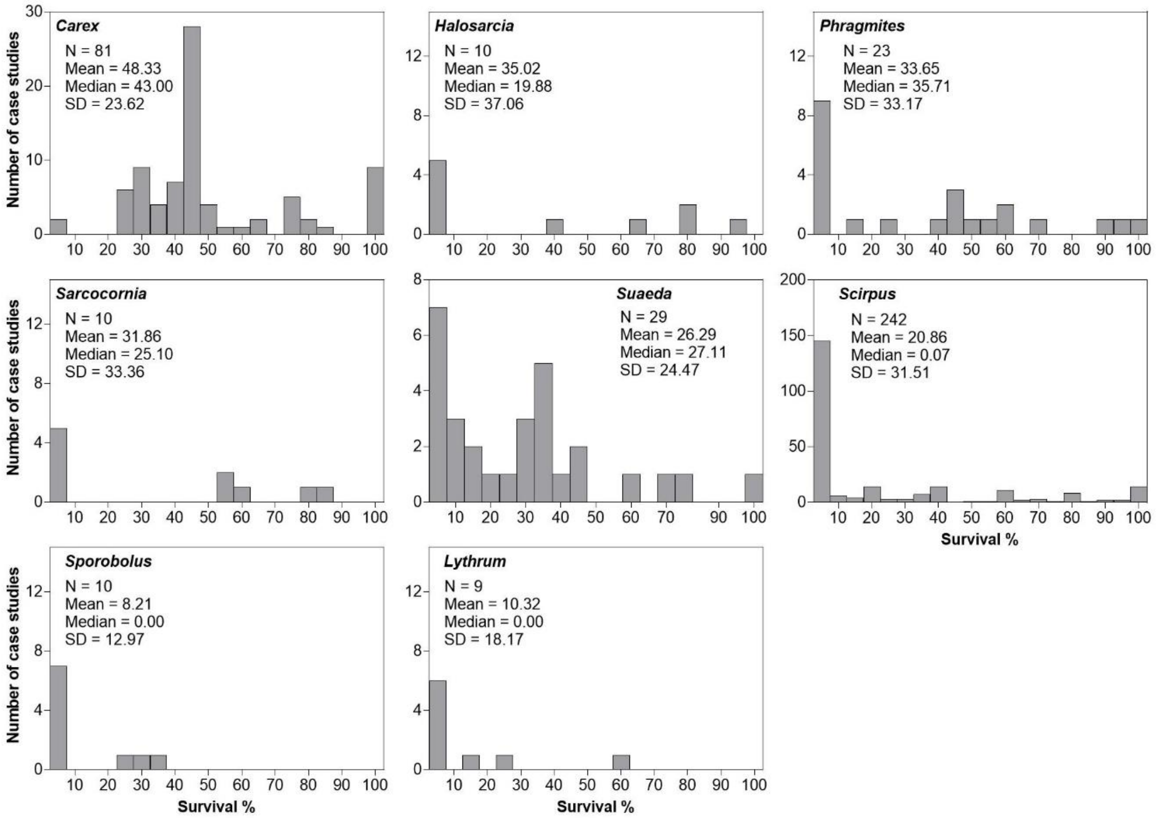

Supplementary Figure 4. Frequency distribution of survival of species with survival rates lower than the mean value (52.8%). Median values and observations are indicated by numbers. Source data are provided as a Source Data file.

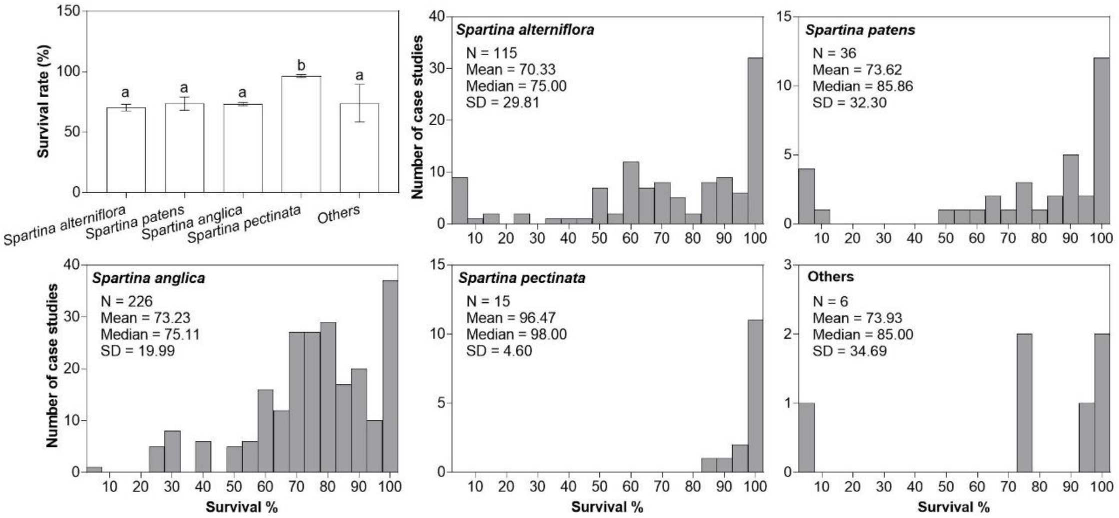

Supplementary Figure 5. Differences in survival rate among *spartina* species and frequency distribution of survival of *spartina* species. Differences in survival rate were assessed using the non-parametric, two-sided Wilcox test, and different letters denote significantly different ($P < 0.05$). Source data are provided as a Source Data file.

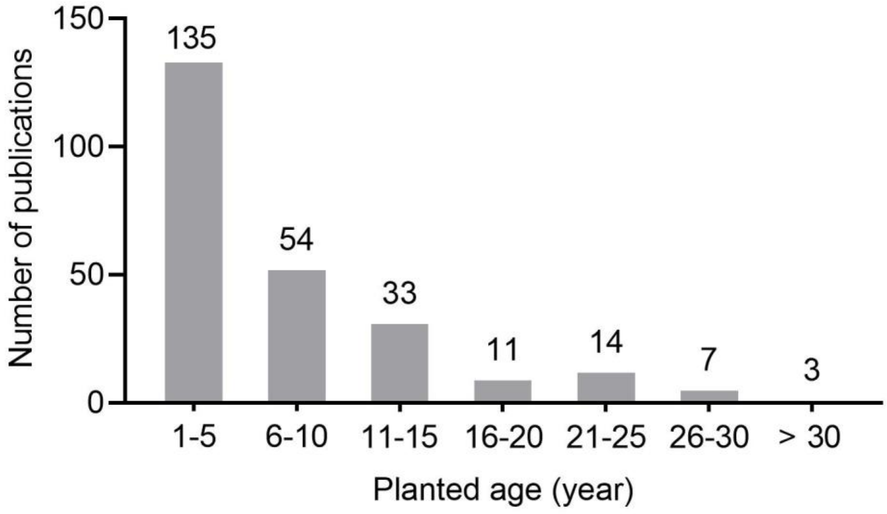

Supplementary Figure 6. Number of publications by planted age. Source data are provided as a Source Data file.

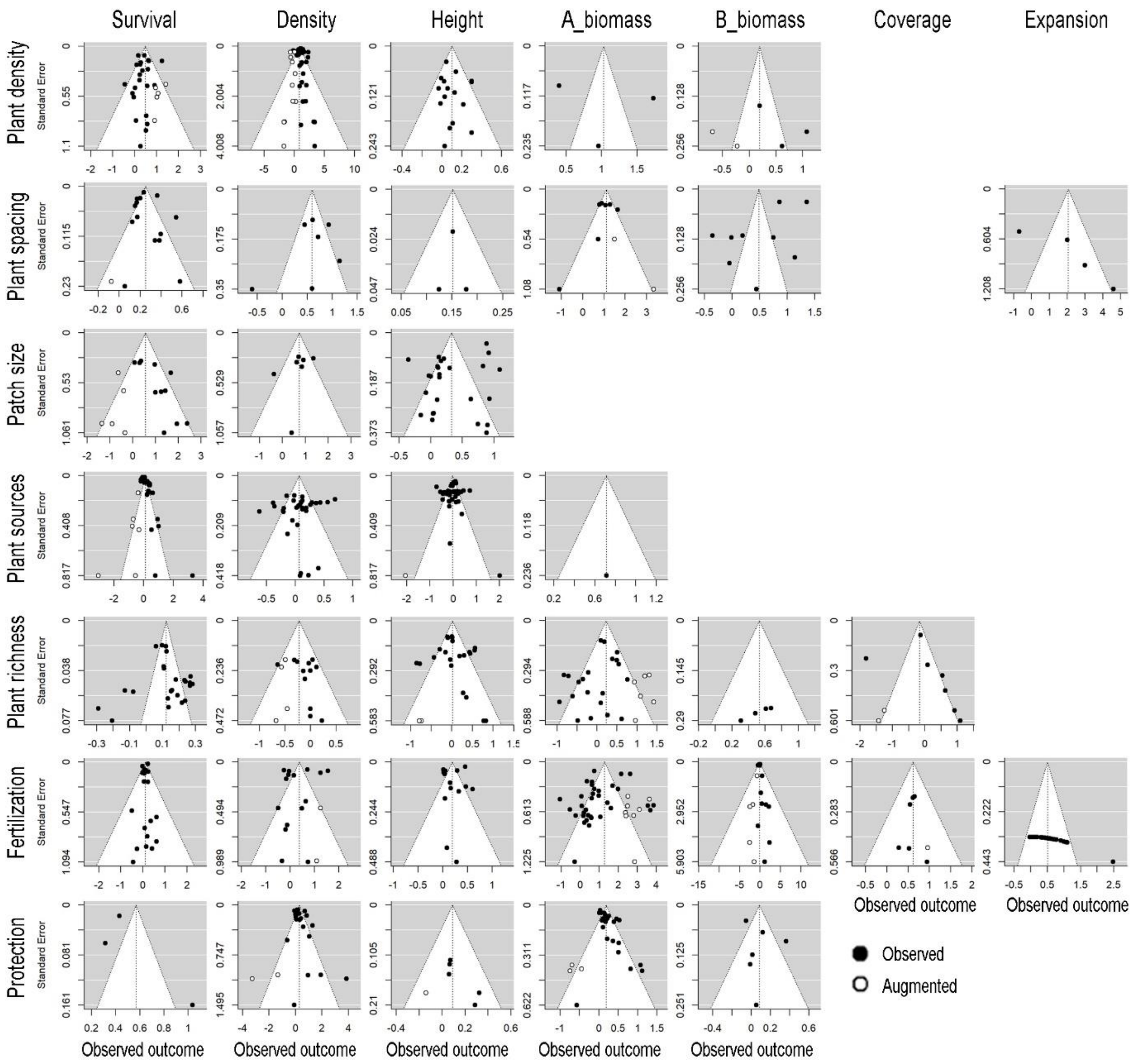

Supplementary Figure 7. Funnel plots for effect size metrics with detected asymmetry. Detailed statistics for publication bias tests are shown in Supplementary Table 5. Source data are provided as a Source Data file.

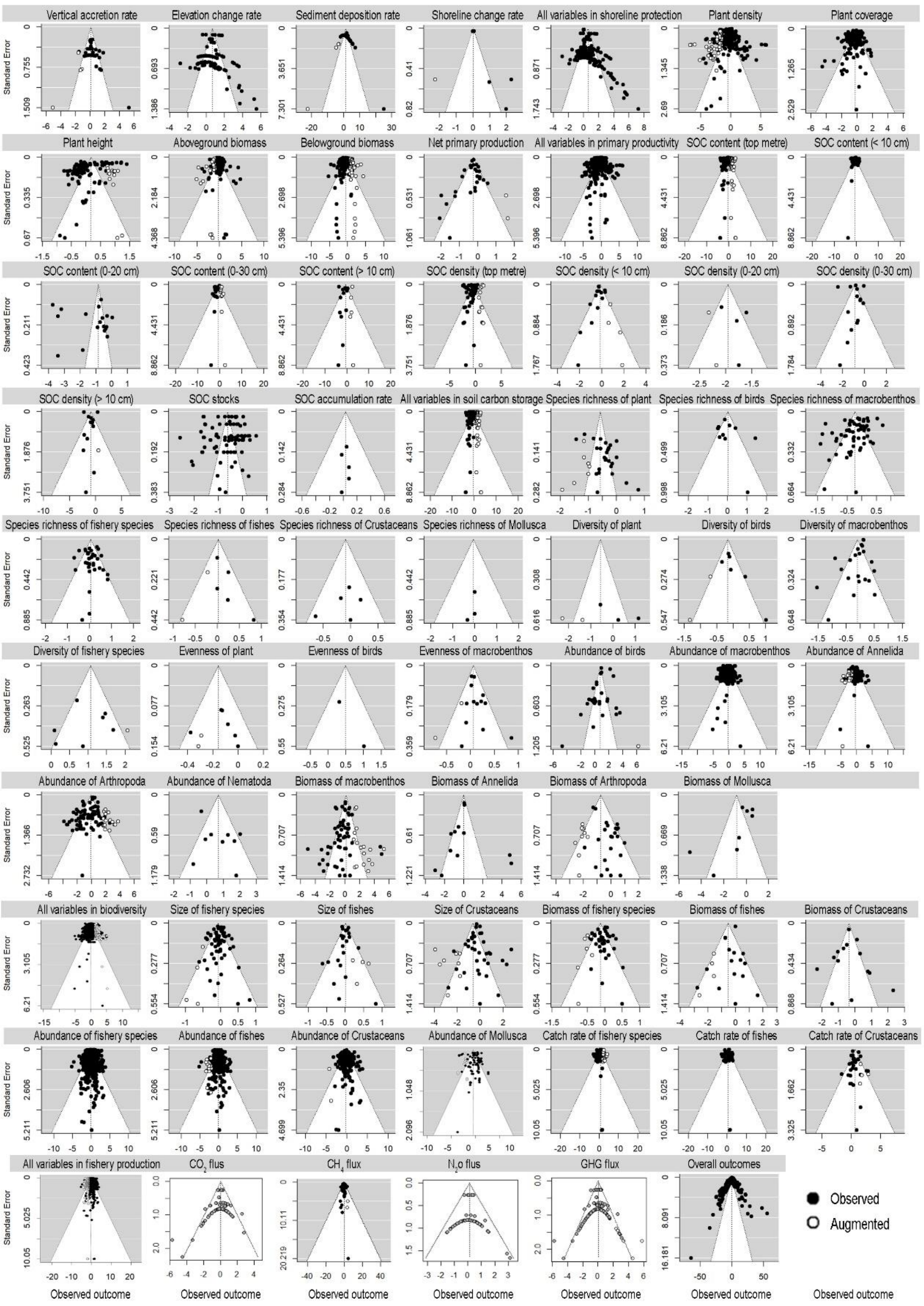

Supplementary Figure 8. Funnel plots for effect size metrics with detected asymmetry compared planted salt marshes with natural wetlands. Detailed statistics for publication bias tests are shown in Supplementary Table 5. Source data are provided as a Source Data file.

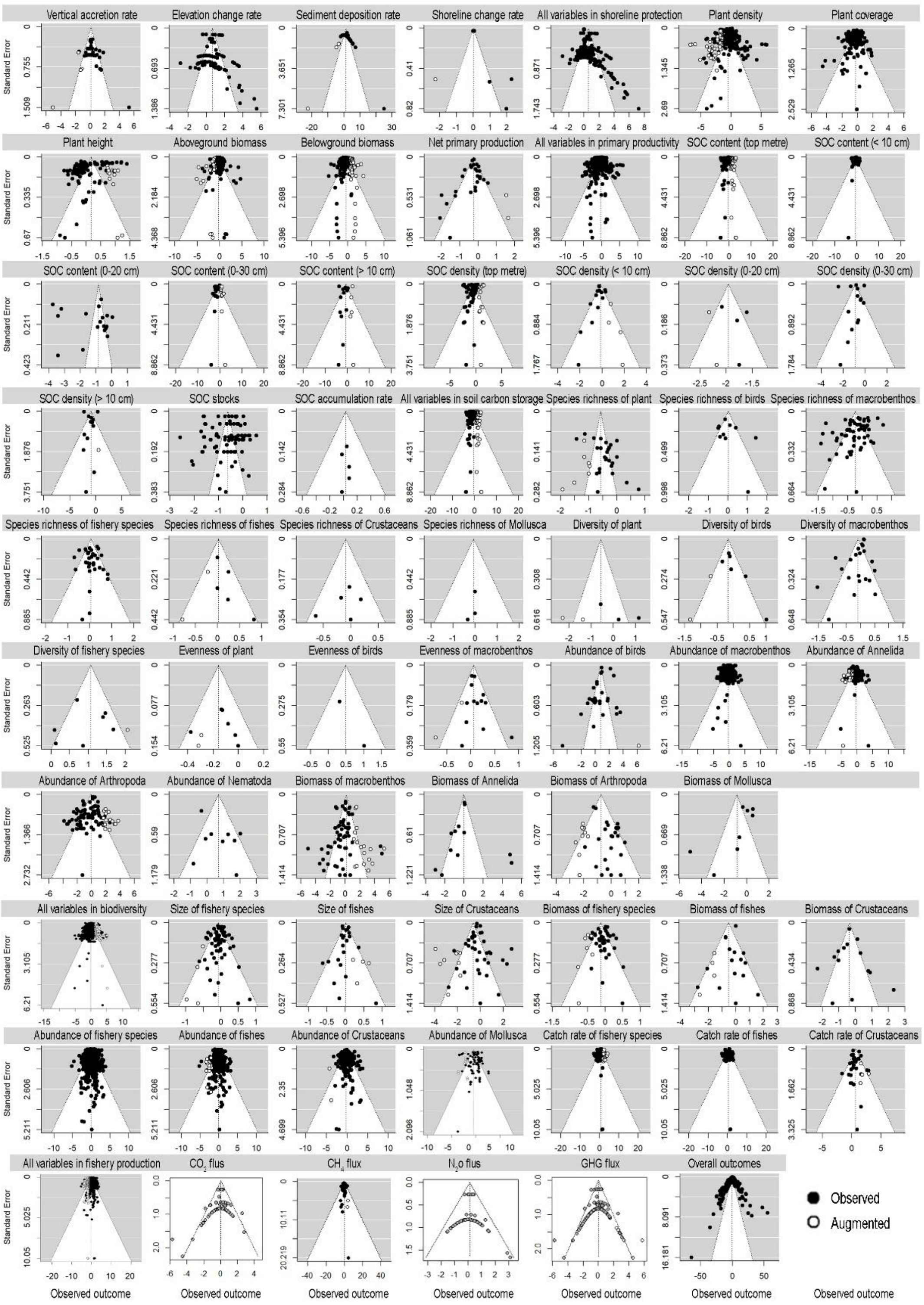

Supplementary Figure 9. Funnel plots for effect size metrics with detected asymmetry compared planted salt marshes with degraded wetlands. Detailed statistics for publication bias tests are shown in Supplementary Table 5. Source data are provided as a Source Data file.

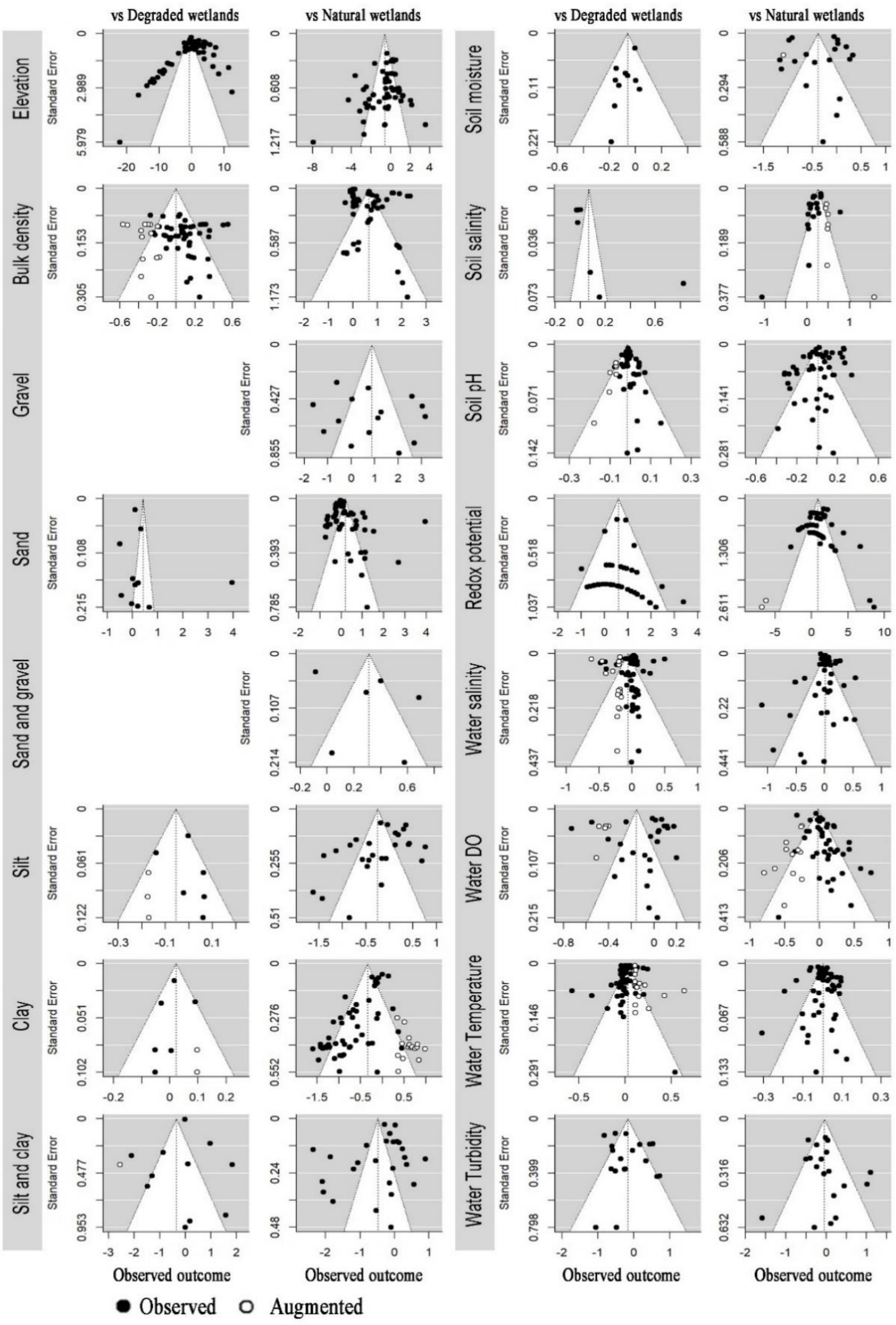

Supplementary Figure 10. Funnel plots for effect size metrics with detected asymmetry for environmental factors. Detailed statistics for publication bias tests are shown in Supplementary Table 5. Source data are provided as a Source Data file.

Supplementary Tables

Supplementary Table 1. Major plant species used in planting of salt marshes. See Supplementary Data 1 for list of articles.

Order	Family	Genus	Species	Type	Plantation sites (reference)
Poales	Poaceae	*Spartina*	*Spartina alterniflora* Loisel.	Perennial	Waquoit Bay National Estuarine Research Reserve, Prudence Island National Estuarine Research Reserve, St. Jones River National Estuarine Research Reserve, Rachel Carson National Estuarine Research Reserve and ACE Basin National Estuarine Research Reserve in the USA (Angus Angermeyer et al., 2018); Roosevelt Inlet of the Delaware Bay in Lewes, Delaware, USA (Balouskus, and Targett, 2012); Lafourche Parish, Louisiana, USA (Baustian et al., 2012); Kiawah Island and Morgan Island in South Carolina, USA (Beck and Gustafson, 2012); Currituck Sound shoreline, North Carolina, USA (Benner et al., 1982); Old Place, Staten Island, Prall’s Island and Gulfport Reach, USA (Bergen et al., 2000); Chesapeake Bay, USA (Bilkovic et al., 2013; Davenport et al., 2018; Palinkas et al., 2018); Neuse River shoreline, USA (Broome et al., 1983); Pine Knoll Shores, USA (Broome et al., 1986); Nags Head, North Carolina, USA (Broome et al., 1988); Barataria Bay, Louisiana, USA (Cagle et al., 2020); Big Egg marsh, Jamaica Bay, New York, USA (Cahoon et al., 2019); Skidaway Island, Georgia, USA (Cain and Cohen, 2014); Dongtai, China (Chung et al., 2004); North Carolina coast, USA (Craft et al., 1988a, 1988b, 1989, 1997 and 2003; Currin et al., 1996, 2008, 2010 and 2017; O'Connor et al., 2011; Polk and Eulie, 2018; Shiau et al., 2016; Shiau et al., 2019; Smith et al., 2018); Waquoit Bay, MA, Prudence Island, RI, Beaufort, NC and Bennett’s Point, SC, USA (Crosby et al., 2017); Newport River Estuary, NC, USA (Davis et al., 2015; Piehler et al., 1998); Sapelo Island, Georgia, USA (Derksen Hooijberg et al., 2018); Turtle Mound in Florida, USA (Donnelly et al., 2017); Tampa bay in Florida, USA (Dontis et al., 2020; Silliman et al., 2015); Trinity island in Louisiana, USA (Feher et al., 2018); Patos Lagoon estuary, Brazil (Freitas et al., 2016); Mosquito Lagoon, Florida, USA (Gaynor et al., 2019; McClenachan et al., 2020; Kibler et al., 2019; Manis et al., 2015; McClenachan et al., 2020; Spiering et al., 2018); Carrot Island, Beaufort, NC, USA (Gittman et al., 2016 and 2018); Barataria Bay in Louisiana, USA (Johnson et al., 2018); Gulf of Mexico in Louisiana, USA (Jones et al., 2019); the coastal United States (Knutson et al., 1981); Mill Brook Marsh, Stratham, New Hampshire, USA (Konisky and Burdick, 2004); Deer Island in Mississippi, USA (Mavrodi et al., 2018); Great Bay Estuary, Maine/New Hampshire, USA (Morgan and Short, 2002); Sapelo creek bank site and Melon Bluff mid marsh in Georgia, USA (Ogburn and Alber, 2006); Calcasieu Lake in Louisiana, USA (Rozas and Minello, 2001); Galveston Bay in Texas, USA (Rozas et al., 2005; Staszak and Armitage, 2013); Little Bay in Mississippi, USA (Sharma et al., 2016); Great Bay Estuary in New Hampshire, USA (Short et al., 2000); Bar Beach Lagoon in New York, Rhode Island, Lavaca Bay in Texas and Hoopers Island

					in Maryland, USA (Society for Ecological Restoration, 2001, 2012 and 2015); Georgetown in South Carolina, USA (Stalter and Batson, 1969); Marineland in Florida, USA (Temmink et al., 2020); Newport River Estuary, North Carolina, USA (Thompson et al., 1995); Caminada Bay, Louisiana, USA (Wilsey et al., 1992); Port Fourchon in Louisiana, USA (Yando et al., 2019)
Poales	Poaceae	*Spartina*	*Spartina patens* (Aiton) Muhl	Perennial	Tampa Bay in Florida, USA (Anastasiou and Brooks, 2003; Dontis et al., 2020); Delaware Bay in Delaware, USA (Balouskus and Targett, 2012 and 2016); Currituck Sound shoreline in North Carolina, USA (Benner et al., 1982); Chesapeake Bay, USA (Bilkovic and Mitchell, 2013; Palinkas et al., 2018); Nags Head in North Carolina, USA (Broome et al., 1988); North Carolina coast, USA (Craft et al., 1988a, 1988b, 1989; Currin et al., 2008 and 2017; Gittman et al., 2016; O'Connor et al., 2011; Shiau et al., 2016; Shiau et al., 2019); Windy Hill Farm and Lynnhaven in VA, USA (Davenport et al., 2018); Trinity island in Louisiana, USA (Feher et al., 2018); Oak Knoll Marsh, Rowley, Massachusetts, USA (Konisky and Burdick, 2004); Deer Island in Mississippi, USA (Mavrodi et al., 2018); Chicken Key in Florida, USA (Milano, 1999); Sabine National Wildlife Refuge in Louisiana, USA (Muench et al., 2019); Little Bay in Mississippi, USA (Sharma et al., 2016); Bar Beach Lagoon in New York, Lavaca Bay in Texas and Hoopers Island in Maryland, USA (Society for Ecological Restoration, 2001 and 2012); Georgetown in South Carolina, USA (Stalter and Batson, 1969); Yancheng Biosphere Reserve in China (Zhou et al., 2003)
Poales	Poaceae	*Spartina*	*Spartina anglica* C. E. Hubbard	Perennial	Red Wharf Bay, UK (Duggan-Edwards et al., 2020); Baarland in The Netherlands (Silliman et al., 2015); De Schorren in the Netherlands (Temmink et al., 2020); Dee Estuary salt marsh, Cheshire, UK (Thompson et al., 1991)
Poales	Poaceae	*Spartina*	*Spartina maritima* (Curtis) Fernald	Perennial	Odiel marshes in Spain (Castillo and Figueroa, 2009; Curado et al., 2012, 2013a, 2013b, 2014a, 2014b, 2014d and 2020)
Poales	Poaceae	*Spartina*	*Spartina foliosa* Trin.	Perennial	Sweetwater Marsh National Wildlife Refuge in San Diego Bay marshes in California, USA (Boyer and Zedler, 1999; Boyer et al., 2000; Zedler, 1993); Western Stege Marsh in California, USA (Foster-Martinez and Variano, 2018); Tijuana Estuary in California, USA (Janousek et al., 2007; O'Brien and Zedler, 2006); Chula Vista Wildlife Reserve in San Diego Bay marshes in California, USA (Zedler, 1993);
Poales	Poaceae	*Spartina*	*Spartina pectinata* Bosc	Perennial	Pokesudie and Shippagan in the province of New Brunswick, Canada (Emond et al., 2016); Pokesudie Island in the Acadian Peninsula of New Brunswick, Canada (Montemayora et al., 2008, 2010 and 2015)
Poales	Poaceae	*Spartina*	*Spartina spartinae* (Trin.) Merr.	Perennial	North end of Key Biscayne and North Miami in Florida, USA (Milano, 1999); Chicken Key, located in the Biscayne Bay Aquatic Preserve, south Biscayne Bay Miami-Dade, Florida, USA (Milano, 1999); Melon Bluff mid marsh in Georgia, USA (Ogburn and Alber, 2006)

Poales	Juncaceae	*Juncus*	*Juncus roemerianus* Scheele	Perennial	Currituck Sound shoreline, North Carolina, USA (Benner et al., 1982); Deer Island in Mississippi, USA (Mavrodi et al., 2018); North end of Key Biscayne and North Miami in Florida, USA (Milano, 1999); Little Bay in Mississippi, USA (Sharma et al., 2016); North Carolina coast, USA (Shiau et al., 2016 and 2019); Grand Bay National Estuarine Research Reserve in Mississippi, USA (Sparks et al., 2013 and 2015)
Poales	Juncaceae	*Juncus*	*Juncus balticus* Willd.	Perennial	Pokesudie Island in the Acadian Peninsula of New Brunswick, Canada (Montemayora et al., 2008, 2010 and 2015)
Poales	Juncaceae	*Juncus*	*Juncus gerardii* Loisel.	Perennial	Oak Knoll Marsh, Rowley, Massachusetts and Mill Brook Marsh, Stratham, New Hampshire, USA (Konisky and Burdick, 2004)
Poales	Juncaceae	*Juncus*	*Juncus effuses* Loisel.	Perennial	Kingman Marsh in Washington, USA (Neff et al., 2009)
Caryophyllales	Amaranthaceae	*Salicornia*	*Salicornia virginica* Loisel.	Perennial	Mugu Lagoon, San Juan Capistrano, California, USA (Armitage et al., 2006); Tijuana Estuary in California, USA (Callaway et al., 1997 and 2003; Zedler et al., 2001 and 2003); Tidal Linkage in Tijuana Estuary in California, USA (Doherty et al., 2011); Fraser estuary, Canada (Pomeroy et al., 1981); Georgetown in South Carolina, USA (Stalter and Batson, 1969)
Caryophyllales	Amaranthaceae	*Salicornia*	*Salicornia bigelovii* Torr.	Annual	Tidal Linkage and the Friendship Marsh in Tijuana Estuary in California, USA (Callaway et al., 2003; Doherty et al., 2011; Zedler et al., 2003); Tijuana Estuary in California, USA (Zedler et al., 2001);
Caryophyllales	Amaranthaceae	*Salicornia*	*Salicornia europaea* Loisel.	Annual	Batiquitos Lagoon in California, USA (Society for Ecological Restoration, 2007a)
Caryophyllales	Amaranthaceae	*Suaeda*	*Suaeda esteroa* Ferren & S. A. Whitmore	Perennial	Tijuana Estuary in California, USA (O'Brien and Zedler, 2006; Zedler et al., 2001);<br>Tidal Linkage in Tijuana Estuary in California, USA (Callaway et al., 2003; Doherty et al., 2011; Zedler et al., 2003); Friendship Marsh in Tijuana Estuary in California, USA (Zedler et al., 2003)
Caryophyllales	Chenopodiaceae	*Suaeda*	*Suaeda australis* (R. Br.) Moq.	Annual	Haslams Creek, Olympics 2000 Site, Sydney, Australia (Burchett et al., 1998; Society for Ecological Restoration, 2007b)
Caryophyllales	Chenopodiaceae	*Suaeda*	*Suaeda salsa* (L.) Pall.	Annual	Liaohe estuary, Chian (Liu et al., 2020)

Poales	Cyperaceae	*Scirpus*	*Scirpus maritimus* (L.) Palla	Perennial	Beninger Slikken and Ventjagersplaten in The Netherlands (Clevering et al., 1997); Eden Estuary, UK (Maynard et al., 2011; Maynard, 2014); Fraser estuary, Canada (Pomeroy et al., 1981); Scheldt Estuary in Groot Buitenschoor, Belgium (Silinski et al., 2016); Eden Estuary in Scotland (Taylor et al., 2019)
Poales	Cyperaceae	*Scirpus*	*Scirpus mariqueter* Tang & F.T.Wang	Perennial	Yangtze estuary, China (Chen et al., 2017, 2019 and 2020; Ge et al., 2019; Hu et al., 2015 and 2017; Tao et al., 2018; Xu et al., 2020)
Poales	Cyperaceae	*Scirpus*	*Scirpus americanus* (Pers.) Volkart	Perennial	Fraser estuary, Canada (Pomeroy et al., 1981)
Poales	Cyperaceae	*Scirpus*	*Scirpus lacustris* (L.) Palla	Perennial	Beninger Slikken and Ventjagersplaten in The Netherlands (Clevering et al., 1997)
Poales	Cyperaceae	*Carex*	*Carex lyngbyei* Hornem.	Perennial	Campbell River estuary in British Columbia, Canada (Dawe et al., 2000); Fraser estuary, Canada (Pomeroy et al., 1981)
Poales	Cyperaceae	*Carex*	*Carex subspathacea* Wormsk. ex Hornem.	Perennial	Hudson Bay, Canada (Handa and Jefferies, 2000)
Poales	Cyperaceae	*Carex*	*Carex paleacea* Schreb. ex Wahlenb.	Perennial	New Brunswick in Pokesudie, Canada (Emond et al., 2016)
Poales	Poaceae	*Puccinellia*	*Puccinellia maritima* (Jacq.) Parl.	Perennial	Nags Head in North Carolina, USA (Broome et al., 1988); Eden Estuary, UK (Maynard et al., 2011; Maynard, 2014)
Poales	Poaceae	*Puccinellia*	*Puccinellia phryganodes* (Trin.) Scribn. and Merr.	Perennial	Hudson Bay, Canada (Handa and Jefferies, 2000)

Poales	Poaceae	*Phragmites*	*Phragmites australis* (Cav.) Trin. ex Steud.	Perennial	Currituck Sound shoreline in North Carolina, USA (Benner et al., 1982); Hangzhou Bay, China (Chen et al., 2020); Oak Knoll Marsh, Rowley, Massachusetts and Mill Brook Marsh, Stratham, New Hampshire, USA (Konisky and Burdick, 2004); Nakdong Estuary, South Korea (Lee et al., 2011); Eden Estuary, UK (Maynard, 2014)
Caryophyllales	Amaranthaceae	*Sarcocornia*	*Sarcocornia quinqueflora* (Bunge ex Ung. - Sternb) A. J. Scott	Perennial	Haslams Creek, Olympics 2000 Site, Sydney, Australia (Burchett et al., 1998; Society for Ecological Restoration, 2007); Homebush Bay, Australia (Santini et al., 2019)
Caryophyllales	Amaranthaceae	*Sarcocornia*	*Sarcocornia perennis* (Mill.) A.J. Scott	Perennial	Odiel marshes in Spain (Curado et al., 2013b, 2014a, 2014c and 2020)
Poales	Typhaceae	*Typha*	*Typha angustifolia* Loisel.	Perennial	Oak Knoll Marsh, Rowley, Massachusetts and Mill Brook Marsh, Stratham, New Hampshire, USA (Konisky and Burdick, 2004);
Poales	Typhaceae	*Typha*	*Typha latifolia* Loisel.	Perennial	Currituck Sound shoreline, North Carolina, USA (Benner et al., 1982)

Supplementary Table 2. Summary of reasons for failure (survival rate ≤ 10%) in plantation of salt marshes.

Species	Restored sites	Reasons for failure	Literature
Spartina alterniflora Loisel.	Kiawah Island and Morgan Island, South Carolina, USA	Locally collected salt marshes have a higher survival than imported plants.	Beck and Gustafson, 2012
Spartina alterniflora Loisel.	Staten Island in New York, USA	shoreline morphology and wave energy were the primary causes of mortality, with goose predation an important secondary cause;<br>Saltmarsh plants were severely impacted by oil spill	Bergen et al., 2000
Sarcocornia quinqueflora (Bunge ex Ung. - Sternb) A. J. Scott<br>*Suaeda australis* (R. Br.) Moq.<br>*Sporobolus virginicus* (L.) Kunth<br>*Halosarcia pergranulata* (J. Black) Paul G. Wilson<br>*Lampranthus tegens* (F. Muell.) N. E. Br.<br>*Wilsonia backhousei* Hook. f.	Haslams Creek, Olympics 2000 Site, Sydney, Australia	The autumn/winter transplants had very low survival and growth rates; Intolerance of saltmarsh plants to soil water contents and saline flooding;	Burchett et al. 1998
Scirpus mariqueter	Yangtze estuary, China	High wave force and sediment deposition.	Chen et al., 2019 and 2020
Spartina alterniflora Loisel.	Erfenshui and Chengmengkou, Dongtai, China	It was attributed to the unusually high wave energies; successful germination of dry seeds was 0;	Chung et al., 2004
Scirpus lacustris	Ventjagersplaten in the Netherlands	Grazed by mute swans (Cygnus olor L.) during three subsequent growing seasons resulted in the complete disappearance of *S. lacustris*	Clevering et al., 1997
Spartina alterniflora Loisel.	Bennett's Point in South Carolina, USA	Decreased survival was observed in northern plants moved to southern marshes, and northern plants moved to the south were less successful	Crosby et al., 2017
Carex lyngbyei	Campbell River estuary, British Columbia, Canada	The higher inundating water salinity and longer inundation periods on these two low-elevation transects contributed to the failure of the vegetation growth.	Dawe et al., 2000

Scirpus mariqueter Tang & F.T.Wang	Yangtze estuary, China	Seed germination rate was extremely low in the field situation; the wave movement easily flushed the seedlings; most of the newly grown seedlings were deeply buried by the sediments.	Ge et al., 2019
Scirpus mariqueter Tang & F.T.Wang	Yangtze estuary, China	High sedimentary rates and tidal wave energy during the early growing season failed to support the establishment of *S. mariqueter*	Hu et al., 2015 and 2017
Juncus gerardii<br>*Typha angustifolia*<br>*Lythrum salicaria*	Oak Knoll Marsh, Rowley, Massachusetts and Mill Brook Marsh, Stratham, New Hampshire, USA	Reduction in halophyte growth was largely due to increased flood duration; brackish species were most reduced by increased salinity.	Konisky and Burdick, 2004
Suaeda salsa	Liaohe estuary, China	The death of planted *S. salsa* was primarily driven by crab herbivory, followed by abiotic stresses (low soil moisture and high salinity) in the high restored marsh.	Liu et al., 2020
Phragmites australis<br>*Scirpus maritimus*<br>*Puccinellia maritima*	Eden Estuary, UK	Seed germination did not occur for either *Scirpus maritimus* or *P. australis*. Further analysis of seed metabolic activity showed that the seeds of *Scirpus maritimus* or *P. australis* were not viable;<br>Seeds were washed away or devoured by benthic invertebrates;<br>The tidal regime within an estuary is a critical factor during natural saltmarsh establishment, mainly due to salinity and flooding;<br>fewer sprigs from the autumn planting session survived;	Maynard, 2014
Scirpus americanus<br>*Carex lyngbyei*<br>*Scirpus maritimus*<br>*Salicornia virginica*<br>*Distichlis spicata*	Fraser estuary, Canada	Higher salinities were responsible for the mortalities;<br>*Carex lyngbyei* plants were heavily grazed by water fowl;	Pomeroy et al., 1981
Scirpus maritimus	Scheldt Estuary, Belgium	Erosion stress influences seedling survival on tidal flats;<br>Survival of transplants was more successful on the tidal flat of the sheltered site compared to the tidal flat of the exposed site;<br>Decreasing survival chances from patches, to rhizome-grown shoots, to seedlings, i.e., a decreasing survival chance with overall size.	Silinski et al., 2016
Borrichis frulescens<br>*Limonium carolinianum*<br>*Spartina alterniflora* Loisel.<br>*Spartina patens* (Aiton) Muhl	Georgetown in South Carolina, USA	The more saline conditions of the High low marsh appear to inhibit the growth of the tall *S. alterniflora*;<br>Neither the dwarf nor tall forms of *S. alterniflora* survived when moved to the High high marsh;<br>*Limonium* may not be as flood tolerant as *Salicornia;*	Stalter and Batson, 1969

		Borrichia might be limited by the effect of ion population (types of ions), and chlorinity; the duration and depth of flooding; the xeric conditions of the High high marsh;	
Spartina alterniflora Loisel.<br>*Spartina anglica* C. E. Hubbard	Marineland in Florida, USA;<br>De Schorren, the Netherlands	High waves or sediment mobility.	Temmink et al., 2020
Batis maritima<br>*Jaumea carnosa*<br>*Salicornia bigelovii*	Friendship Marsh in California, USA	Multiple stresses caused plant mortality: high salinity, sediment deposition, algal smothering and animal activity.	Zedler et al., 2003
Suaeda esteroa	Friendship Marsh in California, USA	Repeated sedimentation events covered leaves, and all plants experienced steady mortality.	Zedler et al., 2008
Spartina patens (Aiton) Muhl	Tanggu Beach, China	As most of the seedlings were buried under sand by accident, very few shoots survived.	Zhou et al., 2003

Supplementary Table 3. Summary of methods to improve the survival rate of plantation in salt marshes.

Species	Restored sites	Methods and seasons	Literature
Spartina alterniflora Loisel.	Kiawah Island and Morgan Island, South Carolina, USA	Locally collected *S. alterniflora* plants had higher survivorship, aboveground biomass, and cumulative stem length than plants from non-local sources.	Beck and Gustafson, 2012
Spartina alterniflora Loisel.	Neuse River shoreline in North Carolina, USA	Survival of plants in the Osmocote treatment was significantly better than any other. This was attributed to the rapid growth of the plants immediately after transplanting which enabled them to become firmly established and able to survive wave action.	Broome et al., 1983
Spartina alterniflora Loisel.	Pine Knoll Shores in North Carolina, USA	The closer-spaced transplants produced higher survival rate and more dry weight per unit area.<br>Closer Plant spacing can reduce wave energy and erosion.	Broome et al., 1986
Scirpus mariqueter Tang & F.T.Wang	Yangtze estuary, China	Increasing size of the transplanted patches could significantly enhance the survival rate; The seed emergence rate with manual support was significantly higher than that of the direct sowing.	Chen et al., 2020
Scirpus lacustris<br>Scirpus maritimus	Beninger Slikken and Ventjagersplaten, the Netherlands	Support of shoots, by wire-netting, could counteract shoot damage by wave action;<br>Plantings were fenced off using wire netting with mesh width of 12 cm can protect the experiment from waterfowl.	Clevering et al., 1997
Spartina alterniflora Loisel.	Bennett's Point in South Carolina, USA	Locally collected salt marshes have a higher survival than imported plants.	Crosby et al., 2017
Spartina anglica C. E. Hubbard	Red Wharf Bay, UK	At high exposure, survival was enhanced by dense planting, which diverted energy away from the vegetation.	Duggan-Edwards et al., 2020
Spartina alterniflora Loisel.	Sapelo Island, Georgia, USA	Co-transplanted mussels locally increased nutrients and reduced sulphide stress, thereby increasing cordgrass growth.	Derksen-Hooijberg et al., 2018
Puccinellia phryganodes<br>*Carex subspathacea*	Hudson Bay, Canada	Treated with fertilizer and peat showed significantly higher growth than those in bare soil. Soils in degraded sites lacked an organic layer, and had significantly higher bulk densities, clay content, surface temperatures and evaporation rates than soils in sites where swards were intact.	Handa and Jefferies, 2000
Scirpus mariqueter Tang & F.T.Wang	Yangtze estuary, China	Seedling survival rates under high planting density were significantly higher than those under medium density and low density.	Hu et al., 2015
Phragmites australis<br>*Scirpus maritimus*	Eden Estuary, UK	Seedling survival rates under high planting density were significantly higher than those under low density.	Maynard, 2014

Puccinellia maritima			
Batis maritima L.<br>*Frankenia salina* (Molina) I.M. Johnston<br>*Jaumea carnosa* (Less.) Gray<br>*Limonium californicum* (Boiss.) Heller<br>*Suaeda esteroa* (Ferren & Whitmore)<br>*Spartina foliosa* Trin.	Tijuana River National Estuarine Research Reserve in California, USA	Survivorship of kelp compost treated plantings increased, because kelp compost significantly increased soil organic matter, total Kjeldahl nitrogen and inorganic nitrogen, and decreased bulk density compared to control plots;<br>Planting seedlings 10-cm apart in tight clusters on the marsh plain increased survivorship compared to 90-cm apart in loose clusters;<br>Tidal creek networks increased survivorship of *Batis maritima* and *Jaumea carnosa.*	O'Brien and Zedler, 2006
Spartina alterniflora Loisel.	Tampa bay in Florida, USA	That clumped configuration increases success of transplanted grasses is likely due in part due to alleviation of anoxia stress by closely planted propagules.	Silliman et al., 2015
Spartina anglica C. E. Hubbard	Baarland in The Netherlands	In the Netherlands study site, reduced edge erosion stress was likely a more important mechanism underlying the emergence of facilitation in clumped configurations.	Silliman et al., 2015
Borrichis frulescens<br>*Limonium carolinianum*<br>*Spartina alterniflora* Loisel.<br>*Spartina patens* (Aiton) Muhl	Georgetown in South Carolina, USA	Locally collected salt marshes have a higher survival than imported plants.	Stalter and Batson, 1969
Spartina alterniflora Loisel.<br>*Spartina anglica* C. E. Hubbard	Marineland in Florida, USA;<br>De Schorren, the Netherlands	Mimicking key emergent traits that locally suppress physical stress using biodegradable establishment structures	Temmink et al., 2020

Supplementary Table 4. Summary of the differences in survival between genera were assessed using the non-parametric, two-sided Wilcox test.

	Puccinellia	*Juncus*	*Batis*	*Salicornia*	*Spartina*	*Frankenia*	*Typha*	*Limonium*	*Jaumea*	*Carex*	*Sarcocornia*	*Halosarcia*	*Phragmites*	*Suaeda*	*Scirpus*	*Sporobolus*
Puccinellia																
Juncus	0.999999															
Batis	0.999999	0.999999														
Salicornia	0.999999	0.999999	0.999999													
Spartina	0.960708	0.999999	0.999999	0.999999												
Frankenia	0.999743	0.999999	0.999999	0.999999	0.999999											
Typha	0.700368	0.946548	0.980855	0.994927	0.962009	0.998928										
Limonium	0.016158	0.300756	0.544847	0.779154	0.097387	0.814281	0.999999									
Jaumea	0.063656	0.446634	0.667320	0.848509	0.276347	0.890502	0.999999	0.999999								
Carex	0.000001	0.002594	0.032842	0.172852	0.000001	0.096108	0.999571	0.999998	0.999999							
Sarcocornia	0.000048	0.002413	0.009277	0.031996	0.000338	0.025438	0.740900	0.824495	0.866839	0.933468						
Halosarcia	0.000259	0.008116	0.025907	0.073965	0.001711	0.065812	0.886006	0.947063	0.962475	0.990998	0.999900					
Phragmites	0.000001	0.000027	0.000455	0.004782	0.000001	0.001505	0.579056	0.599871	0.705604	0.679366	0.999900	0.999900				
Suaeda	0.000001	0.000001	0.000002	0.000080	0.000001	0.000006	0.107500	0.052896	0.112900	0.021840	0.999900	0.999840	0.999900			
Scirpus	0.000001	0.000001	0.000001	0.000001	0.000001	0.000001	0.002622	0.000029	0.000545	0.000001	0.998400	0.975700	0.867400	0.999880		
Sporobolus	0.000001	0.000001	0.000001	0.000004	0.000001	0.000001	0.005000	0.002300	0.005100	0.001700	0.882700	0.735800	0.540300	0.931700	0.992200	
Lythrum	0.000001	0.000001	0.000002	0.000024	0.000001	0.000008	0.014400	0.009400	0.017000	0.009300	0.955500	0.868000	0.750300	0.984600	0.999500	0.999999

Supplementary Table 5. Summary of the differences of environment factors in restored, natural and degraded salt marshes.

Variables	△ restored – natural					△ restored – degraded				
	n	Min., Max.	Median	Mean	SE	n	Min., Max.	Median	Mean	SE
Elevation (cm)	101	-66.00, 39.78	-3.90	-5.53	1.74	83	-184.40, 91.50	0.00	-10.35	5.49
Soil bulk density (g/m^3)	82	-0.30, 1.22	0.54	0.51	0.05	62	-0.29, 0.36	0.05	0.04	0.02
Gravel (%)	15	-2.37, 39.82	1.58	6.07	2.78	0				
Sand (%)	77	-34.30, 86.32	1.66	6.08	2.74	31	-51.15, 86.32	0.85	1.43	3.88
Sand and gravel (%)	10	-8.00, 47.13	8.90	14.88	6.22	4	-2.50, 11.20	4.90	4.63	2.87
Silt (%)	38	-40.00, 16.00	-1.74	-3.68	2.03	18	-5.64, 9.20	-0.16	0.28	0.82
Clay (%)	62	-44.00, 10.2	-13.58	-13.83	1.63	18	-4.00, 5.17	-0.65	-0.53	0.51
Silt and clay (%)	36	-82.13, 33.11	-2.32	-8.51	3.74	129	-86.32, 56.90	-1.00	-1.60	2.00
Soil Moisture (%)	28	-74.70, 8.00	-26.50	-27.97	5.02	19	-25.60, 14.64	-2.94	-3.13	1.85
Soil porewater salinity (ppt)	21	-9.27, 28.00	8.20	11.12	2.42	10	-3.00, 0	-0.50	-1.00	0.37
Soil porewater pH	60	-2.02, 2.60	0.20	0.35	0.15	44	-1.18, 1.03	-0.06	0.00	0.06
Soil Redox potential (Eh) (mV)	55	-118.00, 375.39	84.76	88.47	14.37	44	-78.02, 226.81	38.00	44.37	9.47
Surface water salinity (ppt)	89	-9.40, 3.93	-0.10	-0.96	0.29	64	-5.00, 4.90	0.06	0.10	0.18
Surface water dissolved oxygen (mg/L)	65	-1.90, 2.67	0.23	0.30	0.11	34	-7.00, 2.90	-0.15	-0.45	0.36
Surface water temperature (℃)	95	-6.25, 8.72	0.20	0.13	0.20	77	-9.09, 6.10	-0.10	-0.15	0.25
Surface water turbidity (FTU)	27	-66.30, 42.70	0.00	-0.26	3.23	27	-57.60, 16.40	-6.40	-7.86	2.92

Supplementary Table 6. Summary of random-effects model effect size estimates and publication bias tests examining the effect sizes of factors selected on different plant performance measures. The Cochran's Q statistic test was used to test whether effect sizes were homogeneous across studies. Rosenthal's fail-safe number and funnel plot trimfill method were used to test publication bias. Results are considered robust if fail-safe numbers are greater than 5n + 10, where n is the number of studies included in the meta-analysis. *P* values reflect two-sided tests. Significant *P* values (< 0.05) are shown in bold.

Factors	Effect size metrics	Random-effects model				Homogeneity test			Fail-safe number test		Trimfill			
		n	estimate	95% CIs	*P*	Cochran's Q	df	*P*	FSN (5n + 10)	*P*	*n* added	estimate	95% CIs	*P*
Plant density (high VS low)	Survival	26	0.406	0.247, 0.564	**0.00001**	53.957	25	**0.0007**	530 (140)	**0.0001**	7	0.4885	0.3378, 0.6392	**0.0001**
	Density	32	1.052	0.793, 1.31	**0.00001**	160.278	31	**0.0001**	5270 (170)	**0.0001**	11	0.8349	0.5563, 1.1135	**0.0001**
	Height	16	0.102	0.041, 0.164	**0.001**	20.702	15	0.147	83 (90)	**0.0001**	0			
	Aboveground biomass	3	1.03	0.25, 1.81	**0.0096**	75.659	2	**0.0001**	186 (25)	**0.0001**	0			
	Belowground biomass	3	0.61	0.088, 1.133	**0.022**	11.197	2	**0.004**	25 (25)	**0.0001**	2	0.1935	-0.4113, 0.7984	0.5306
plant spacing (tight VS loose)	Survival	14	0.26	0.19, 0.329	**0.000001**	79.247	13	**0.0001**	2328 (80)	**0.0001**	1	0.2526	0.1832, 0.322	**0.0001**
	Density	7	0.599	0.265, 0.932	**0.0004**	24.698	6	**0.0004**	238 (45)	**0.0001**	0			
	Height	3	0.152	0.118, 0.185	**0.0001**	0.648	2	0.723	70 (25)	**0.0001**	0			
	Expansion	4	0.6	-0.064, 1.264	0.076	24.65	3	**0.0001**	62 (30)	**0.0001**	0			
	Aboveground biomass	7	1.065	0.799, 1.332	**0.0001**	16.93	6	**0.0095**	388 (45)	**0.0001**	2	1.1187	0.8592, 1.3783	**0.0001**
	Belowground biomass	9	0.485	0.097, 0.873	**0.014**	417.98	8	**0.0001**	2349 (55)	**0.0001**	0			
Patch size (large VS small)	Survival	11	0.922	0.501, 1.344	**0.0001**	20.052	10	**0.029**	155 (65)	**0.0001**	5	0.5647	0.1165, 1.013	**0.0135**
	Density	7	0.738	0.36, 1.115	**0.0001**	12.29	6	0.056	77 (45)	**0.0001**	0			
	Height	26	0.3296	0.1655, 0.4938	**0.0001**	336.355	25	**0.0001**	1772 (140)	**0.0001**	0			
Patch sources (local VS nonlocal)	Survival	43	0.162	0.09, 0.234	**0.0001**	787.074	42	**0.0001**	1547 (225)	**0.0001**	7	0.1226	0.0466, 0.1985	**0.0016**
	Density	32	0.069	-0.034, 0.171	0.188	155.061	31	**0.0001**	91 (170)	**0.0007**	0			
	Height	41	-0.018	-0.106, 0.071	0.698	202.355	40	**0.0001**	0 (215)	0.3569	1	-0.0233	-0.1126, 0.0659	0.6085
	Aboveground biomass	1	0.713	0.2507, 1.1754	**0.0025**	0	0	1	3 (15)	**0.0013**	0			
plant richness (multispecies VS one)	Survival	24	0.123	0.066, 0.181	**0.0001**	167.639	23	**0.0001**	1955 (130)	**0.0001**	0			
	Density	12	-0.14	-0.287, 0.007	0.062	10.9821	11	0.4448	2 (70)	**0.0387**	4	-0.2231	-0.363, -0.0832	**0.0018**
	Height	22	0.043	-0.118, 0.204	0.604	73.397	21	**0.0001**	0 (120)	0.1448	2	0.0187	-0.1409, 0.1782	0.8188
	Coverage	7	0.118	-0.631, 0.868	0.757	70.949	6	**0.0001**	0 (45)	0.1694	2	-0.1632	-0.8568, 0.5305	0.6448
	Aboveground biomass	21	0.007	-0.21, 0.225	0.946	46.844	20	**0.0006**	0 (115)	0.3008	6	0.2286	-0.02, 0.4773	0.0715
	Belowground biomass	4	0.532	0.272, 0.793	**0.0001**	1.021	3	0.796	20 (30)	**0.0001**	0			
Fertilization (fertilized	Survival	20	0.114	0.037, 0.191	**0.004**	38.272	19	**0.005**	144 (110)	**0.0001**	0			
	Density	15	0.311	-0.039, 0.662	0.082	371.339	14	**0.0001**	469 (85)	**0.0001**	2	0.3778	0.0407, 0.7149	**0.0281**
	Height	15	0.191	0.086, 0.296	**0.0003**	208.034	14	**0.0001**	717 (85)	**0.0001**	0			

VS unfertilized)	Coverage	6	0.602	0.382, 0.822	**0.0001**	0.957	5	0.966	47 (40)	**0.0001**	1	0.6199	0.4055, 0.8343	**0.0001**
	Expansion	32	0.5089	0.3722, 0.6457	**0.0001**	46.4615	31	**0.0367**	1021 (170)	**0.0001**	0			
	Aboveground biomass	34	0.966	0.577, 1.355	**0.0001**	318.386	33	**0.0001**	3375 (180)	**0.0001**	8	1.2852	0.9004, 1.67	**0.0001**
	Belowground biomass	14	-0.102	-0.242, 0.038	0.154	7.022	13	0.901	0 (80)	0.4019	5	-0.1104	-0.2497, 0.0289	0.1203
Protective structures (Protection VS control)	Survival	3	0.5701	0.1572, 0.9831	**0.0068**	17.8086	2	**0.0001**	488 (25)	**0.0001**	0			
	Density	22	0.321	0.161, 0.481	**0.0001**	98.2	21	**0.0001**	584 (120)	**0.0001**	2	0.295	0.1313, 0.4587	**0.0004**
	Height	5	0.12	-0.007, 0.247	0.063	2.37	4	0.668	4 (35)	**0.0175**	1	0.0918	-0.028, 0.2116	0.1331
	Aboveground biomass	25	0.209	0.14, 0.279	**0.0001**	87.86	24	**0.0001**	1010 (135)	**0.0001**	3	0.1918	0.1211, 0.2625	**0.0001**
	Belowground biomass	6	0.087	-0.056, 0.229	0.234	19.891	5	**0.0013**	3 (40)	**0.0271**	0			

Supplementary Table 7. Summary of random-effects model effect size estimates and publication bias tests examining the effect sizes of planted salt marshes when compared with natural salt marshes. Detailed test statistics are presented in Supplementary Table 5. *P* values reflect two-sided tests. Significant *P* values (< 0.05) are shown in bold.

Effect size metrics	Random-effects model				Homogeneity test			Fail-safe number test		Trimfill			
	n	estimate	95% CIs	*P*	Cochran's Q	df	*P*	FSN (5n + 10)	*P*	*n* added	estimate	95% CIs	*P*
All outcomes	3832	-0.2548	-0.290, -0.220	**0.0001**	16024.26	3831	**0.0001**	3716326 (19170)	**0.0001**	0			
Vertical accretion rate	50	0.1686	0.0345, 0.3027	**0.0137**	78.122	49	**0.005**	126 (260)	**0.0006**	4	0.11	-0.0515, 0.2715	0.1817
Elevation change rate	93	0.684	0.453, 0.915	**0.0001**	658.667	92	**0.0001**	8887 (475)	**0.0001**	0			
Sediment deposition rate	35	1.81	1.104, 2.516	**0.0001**	186.04	34	**0.0001**	1643 (185)	**0.0001**	5	1.3594	0.5631, 2.1557	**0.0008**
Shoreline change rate	20	0.0159	0.0023, 0.0295	**0.0217**	37.758	19	**0.006**	94 (110)	**0.0001**	1	0.0155	0.0019, 0.0291	**0.0259**
All variables in shoreline protection	198	0.5969	0.449, 0.751	**0.0001**	1436.683	197	**0.0001**	25279 (1000)	**0.0001**	0			
Plant density	320	-0.201	-0.324, -0.078	**0.0014**	8008.558	319	**0.0001**	50689 (1610)	**0.0001**	46	-0.4766	-0.6186, -0.3346	**0.0001**
Plant coverage	156	-0.2462	-0.3594, -0.133	**0.0001**	1395.142	155	**0.0001**	5670 (790)	**0.0001**	0			
Plant height	99	0.0729	-0.021, 0.167	0.1297	4431.588	98	**0.0001**	18490 (505)	**0.0001**	15	0.1733	0.0773, 0.2692	**0.0004**
Aboveground biomass	359	-0.1289	-0.1834, -0.0745	**0.0001**	3423.1729	358	**0.0001**	39824 (1805)	**0.0001**	36	-0.2139	-0.2743, -0.1536	**0.0001**
Belowground biomass	317	-0.6143	-0.696, -0.5326	**0.0001**	4423.1753	316	**0.0001**	248893 (1595)	**0.0001**	44	-0.4539	-0.5474, -0.3605	**0.0001**
Net primary production	28	-0.266	-0.412, -0.121	**0.0001**	77.112	27	**0.0001**	690 (150)	**0.0001**	2	-0.2355	-0.4184, -0.0527	**0.0116**
All variables in primary productivity	1279	-0.266	-0.309, -0.223	**0.0001**	27184.104	1278	**0.0001**	792826 (6405)	**0.0001**	0			
SOC content (top meter)	235	-0.644	-0.769, -0.519	**0.0001**	6349.512	234	**0.0001**	128486 (1185)	**0.0001**	47	-0.3508	-0.4934, -0.2081	**0.0001**
SOC content (< 10 cm)	136	-0.588	-0.719, -0.457	**0.0001**	2024.941	135	**0.0001**	48264 (690)	**0.0001**	0			
SOC content (0-20 cm)	32	-0.837	-1.259, -0.416	**0.0001**	2298.545	31	**0.0001**	8271 (170)	**0.0001**	0			
SOC content (0-30 cm)	30	-1.087	-1.417, -0.758	**0.0001**	152.49	29	**0.0001**	1602 (160)	**0.0001**	11	-0.7066	-1.0531, -0.36	**0.0001**
SOC content (>10 cm)	15	-1.105	-1.912, -0.299	**0.0001**	119.461	14	**0.0001**	269 (85)	**0.0001**	5	-0.5559	-1.4726, 0.3608	0.2346
SOC density (top meter)	48	-0.803	-1.045, -0.561	**0.0001**	13182.4757	47	**0.0001**	37806 (250)	**0.0001**	15	-0.4505	-0.722, -0.179	**0.0011**
SOC density (<10 cm)	14	-0.197	-0.388, -0.006	**0.043**	41.702	13	**0.0001**	153 (80)	**0.0001**	4	-0.1331	-0.3289, 0.0626	0.1824

SOC density (0-20 cm)	4	-1.826	-2.082, -1.57	**0.0001**	8.414	3	**0.038**	828 (30)	**0.0001**	2	-1.9558	-2.2109, -1.7006	**0.0001**
SOC density (0-30 cm)	15	-0.865	-1.273, -0.456	**0.0001**	1609.505	14	**0.0001**	6019 (85)	**0.0001**	0			
SOC density (>10 cm)	15	-0.918	-1.403, -0.434	**0.0001**	10810.951	14	**0.0001**	5633 (85)	**0.0001**	1	-0.8946	-1.3734, -0.4157	**0.0003**
SOC stocks	87	-0.616	-0.749, -0.483	**0.0001**	7656.494	86	**0.0001**	168646 (445)	**0.0001**	0			
SOC accumulation rate	5	0.034	-0.118, 0.186	0.662	0.306	4	0.989	0 (35)	0.3357	0			
All variables in soil carbon storage	375	-0.64	-0.7302, 0.5533	**0.0001**	28502.1635	374	**0.0001**	928018 (1885)	**0.0001**	69	-0.3918	-0.4928, -0.2907	**0.0001**
CO_2 flux	86	0.0345	-0.1017, 0.1708	0.6195	95.53	85	0.2041	0 (440)	0.2892	0			
CH_4 flux	70	-0.2056	-0.3816, -0.0297	**0.0219**	67.42	69	0.5312	225 (360)	**0.0004**	2	-0.0383	-0.176, 0.1376	**0.0285**
N_2O flux	70	0.1183	-0.0302, 0.2667	0.1184	44.59	69	0.9901	0 (360)	0.0508				
All variables in GHG	226	-0.0111	-0.0988, 0.0765	0.8035	216.6	225	0.6442	0 (1140)	0.095	6	0.0039	-0.084, 0.0917	0.9308
Species richness of vegetation	26	-0.4056	-0.5684, -0.2428	**0.0001**	266.091	25	**0.0001**	2554 (140)	**0.002**	7	-0.5689	-0.7455, -0.3923	**0.0001**
Species richness of birds	10	0.025	-0.33, 0.38	0.891	39.178	9	**0.0001**	0 (60)	0.4418	0			
Species richness of macrobenthos	64	-0.232	-0.339, -0.124	**0.0001**	690.653	63	**0.0001**	2513 (330)	**0.0001**	0			
Species richness of fishery species	37	0.0593	-0.0431, 0.1616	0.2562	92.6989	36	**0.0001**	14 (125)	**0.0268**	0			
Species richness of fishes	5	0.116	-0.074, 0.306	0.2318	4.694	4	0.32	1 (35)	**0.0372**	2	0.0236	-0.1491, 0.1964	0.7885
Species richness of Crustaceans	5	-0.089	-0.326, 0.147	0.4591	3.907	4	0.419	0 (35)	0.188	0			
Species richness of Mollusca	4	-0.057	-0.783, 0.67	0.878	0.111	3	0.991	0 (30)	0.4273	0			
Diversity of vegetation	3	0.2342	-0.768, 1.2364	0.6469	4.8713	2	0.0875	0 (25)	0.2485	2	-0.566	-1.7318, 0.5998	0.3413
Diversity of birds	6	-0.1087	-0.2303, 0.013	0.0799	10.227	5	0.069	0 (40)	0.2773	2	-0.1607	-0.4201, 0.0987	0.2246
Diversity of macrobenthos	22	-0.095	-0.266, 0.076	0.275	110.614	21	**0.0001**	488 (120)	**0.0001**	0			
Diversity of fishery species	7	0.9426	0.4903, 1.3948	**0.0001**	15.438	6	**0.017**	114 (45)	**0.0001**	1	1.0704	0.5954, 1.5453	**0.0001**
Evenness of vegetation	6	-0.129	-0.215, -0.043	**0.0034**	5.249	5	0.386	12 (40)	**0.0025**	2	-0.1565	-0.2356, -0.0773	**0.0001**
Evenness of birds	2	0.493	-0.098, 1.084	0.102	1.329	1	0.249	2 (20)	**0.0135**	0			
Evenness of macrobenthos	13	0.076	0.02, 0.132	**0.0076**	14.615	12	0.263	33 (75)	**0.001**	2	0.0624	0.0077, 0.1171	**0.0253**
Abundance of birds	27	0.638	0.081, 1.195	**0.0247**	684.677	26	**0.0001**	5027 (145)	**0.0001**	1	0.7678	0.1461, 1.3896	**0.0155**
Abundance of macrobenthos	428	-0.292	-0.389, -0.195	**0.0001**	38586.664	427	**0.0001**	31462 (2150)	**0.0001**	0			
Abundance of Annelida	169	-0.182	-0.322, -0.042	**0.011**	598.812	168	**0.0001**	1884 (855)	**0.0001**	22	-0.3814	-0.5385, -0.2243	**0.0001**
Abundance of Arthropoda	87	-0.47	-0.726, -0.213	**0.0003**	381.535	86	**0.0001**	751 (445)	**0.0001**	17	-0.069	-0.3657, 0.2276	0.6484
Abundance of Nematoda	9	0.704	0.036, 1.373	**0.039**	27.026	8	**0.0001**	23 (55)	**0.001**	0			

Biomass of macrobenthos	62	-0.335	-0.649, -0.02	**0.037**	238.682	61	**0.0001**	275 (320)	**0.0001**	17	0.1629	-0.2013, 0.5271	0.3806
Biomass of Annelida	12	-0.02	-1.319, 1.279	0.976	73.887	11	**0.0001**	0 (70)	0.4028	0			
Biomass of Arthropoda	25	-0.232	-0.584, 0.12	0.1966	42.8314	24	**0.0104**	19 (135)	**0.0154**	10	-0.7435	-1.1105, -0.3764	**0.0001**
Biomass of Mollusca	8	-0.856	-2.085, 0.373	0.172	44.615	7	**0.0001**	9 (50)	**0.0085**	0			
All variables in biodiversity conservation	668	-0.166	-0.2299, -0.1021	**0.0001**	43405.038	667	**0.0001**	21714 (3350)	**0.0001**	78	0.0099	-0.640, 0.084	**0.0001**
Size of fishery species	84	-0.063	-0.104, -0.022	**0.003**	288.708	83	**0.0001**	704 (430)	**0.0001**	5	-0.0722	-0.1136, -0.0309	**0.0006**
Size of fishes	22	-0.0022	-0.0522, 0.0477	0.9305	50.1328	21	**0.0003**	0 (125)	0.2652	4	0.0112	-0.0394, 0.0619	0.6635
Size of Crustaceans	61	-0.077	-0.127, -0.027	**0.002**	230.129	60	**0.0001**	592 (315)	**0.0001**	11	-0.1225	-0.175, -0.07	**0.0001**
Biomass of fishery species	64	-0.267	-0.53, -0.005	**0.046**	267.204	63	**0.0001**	1455 (330)	**0.0001**	13	-0.6339	-0.9378, -0.33	**0.0001**
Biomass of fishes	30	-0.307	-0.604, -0.009	**0.044**	48.298	29	**0.014**	29 (160)	**0.0106**	6	-0.5305	-0.851, -0.21	**0.0012**
Biomass of Crustaceans	24	-0.353	-0.804, 0.093	0.1199	128.691	23	**0.0001**	273 (130)	**0.0001**	0			
Abundance of fishery species	746	-0.093	-0.16, -0.025	**0.007**	21225.299	745	**0.0001**	31600 (3740)	**0.0001**	0			
Abundance of fishes	322	-0.0284	-0.1367, 0.0799	0.6072	3548.3662	321	**0.0001**	2134 (1625)	**0.0001**	15	-0.1018	-0.2156, 0.012	0.0794
Abundance of Crustaceans	323	-0.2196	-0.311, -0.128	**0.0001**	1236.953	322	**0.0001**	13109 (1625)	**0.0001**	2	-0.2254	-0.3175, -0.1333	**0.0001**
Abundance of Mollusca	82	-0.205	-0.479, 0.069	0.142	915.45	81	**0.0001**	798 (420)	**0.0001**	13	-0.4655	-0.738, -0.193	**0.0001**
Catch rate of fishery species	147	0.496	0.344, 0.648	**0.0001**	2079.207	146	**0.0001**	43606 (745)	**0.0001**	17	0.7068	0.5275, 0.886	**0.0001**
Catch rate of fishes	82	0.465	0.186, 0.744	**0.001**	1346.149	81	**0.0001**	8384 (420)	**0.0001**	0			
Catch rate of Crustaceans	38	0.599	0.372, 0.826	**0.0001**	109.766	37	**0.0001**	861 (200)	**0.0001**	4	0.6655	0.4395, 0.8915	**0.0001**
All variables in fishery production	1086	-0.036	-0.092, 0.019	0.202	35694.06	1085	**0.0001**	3921 (5440)	**0.0002**	132	-0.2315	-0.2944, -0.1686	**0.0001**

Supplementary Table 8. Summary of random-effects model effect size estimates and publication bias tests examining the effect sizes of planted salt marshes when compared with degraded salt marshes. Detailed test statistics are presented in Supplementary Table 5. *P* values reflect two-sided tests. Significant *P* values (< 0.05) are shown in bold.

Effect size metrics	Random-effects model				Homogeneity test			Fail-safe number test		Trimfill			
	n	estimate	95% CIs	*P*	Cochran's Q	df	*P*	FSN (5n + 10)	*P*	*n* added	estimate	95% CIs	*P*
All outcomes	1698	0.694	0.628, 0.76	**0.0001**	11936.691	1697	**0.0001**	5770860 (8500)	**0.0001**	0			
Vertical accretion rate	58	0.434	0.207, 0.661	**0.0002**	187.299	57	**0.0001**	874 (300)	**0.0001**	0			
Elevation change rate	69	0.605	0.259, 0.952	**0.0006**	350.803	68	**0.0001**	2226 (355)	**0.0001**	0			
Sediment deposition rate	43	0.9696	0.5817, 1.3574	**0.0001**	140.155	42	**0.0001**	1153 (225)	**0.0001**	13	0.4631	0.0553, 0.8709	**0.026**
Shoreline change rate	29	0.487	0.167, 0.806	**0.003**	133.821	28	**0.0001**	549 (155)	**0.0001**	7	0.1201	-0.4198, 0.66	0.6629
All variables in shoreline protection	199	0.611	0.447, 0.774	**0.0001**	963.197	198	**0.0001**	18581 (1005)	**0.0001**	0			
Plant density	40	2.375	1.926, 2.824	**0.0001**	235.511	39	**0.0001**	7743 (210)	**0.0001**	15	1.6761	1.1668, 2.1854	**0.0001**
Plant coverage	91	1.926	1.624, 2.227	**0.0001**	1121.395	90	**0.0001**	51150 (465)	**0.0001**	25	1.502	1.2055, 1.7984	**0.0001**
Plant height	37	0.923	0.657, 1.188	**0.0001**	644.617	36	**0.0001**	14048 (195)	**0.0001**	0			
Aboveground biomass	59	1.062	0.689, 1.436	**0.0001**	656.958	58	**0.0001**	8599 (305)	**0.0001**	0			
Belowground biomass	45	1.462	1.138, 1.786	**0.0001**	45627.262	44	**0.0001**	17592 (235)	**0.0001**	0			
All variables in primary productivity	272	1.549	1.384, 1.714	**0.0001**	48915.39	271	**0.0001**	434149 (1370)	**0.0001**	55	1.1393	0.9547, 1.3238	**0.0001**
SOC content (top meter)	72	0.145	0.041, 0.249	**0.006**	848.183	71	**0.0001**	1891 (370)	**0.0001**	0			
SOC content (< 10 cm)	42	0.189	0.022, 0.355	**0.0267**	720.726	41	**0.0001**	808 (220)	**0.0001**	4	0.2632	0.0922, 0.4341	**0.0026**
SOC content (0-20 cm)	4	0.227	-0.225, 0.678	0.3251	30.122	3	**0.0001**	13 (30)	**0.0004**	0			
SOC content (0-30 cm)	9	0.069	-0.22, 0.357	0.64	82.385	8	**0.0001**	48 (55)	**0.0001**	1	0.0421	-0.2465, 0.3308	0.7748
SOC density (top meter)	18	0.174	0.063, 0.284	**0.002**	23.813	17	0.125	73 (100)	**0.0001**	2	0.2077	0.0974, 0.3181	**0.0002**
SOC stocks	9	0.725	0.344, 1.107	**0.0002**	165.264	8	**0.0001**	798 (55)	**0.0001**	0			
All variables in soil carbon storage	99	0.2131	0.1218, 0.3044	**0.0001**	1107.0364	98	**0.0001**	6656 (505)	**0.0001**	16	0.3114	0.2182, 0.4045	**0.0001**
Species richness of vegetation	20	0.8672	0.4942, 1.2401	**0.0001**	128.3264	19	**0.0001**	1580 (110)	**0.0001**	3	0.6856	0.2372, 1.134	**0.0027**
Species richness of macrobenthos	22	0.126	0.005, 0.247	**0.041**	138.032	21	**0.0001**	160 (120)	**0.0001**	0			
Species richness of fishery species	17	0.5509	0.2634, 0.8383	**0.0002**	121.2813	16	**0.0001**	824 (95)	**0.0001**	0			
Species richness of fishes	3	-0.009	-0.642, 0.624	0.978	11.624	2	**0.003**	0 (25)	0.4867	0			
Species richness of Crustaceans	3	0.489	0.15, 0.829	**0.005**	2.88	2	0.237	10 (25)	**0.0004**	0			
Species richness of Mollusca	3	0.842	-0.262, 1.946	0.135	2.035	2	0.362	1 (25)	**0.0474**	2	0.0001	-1.2612, 1.2612	1
Diversity of macrobenthos	19	0.149	-0.072, 0.37	0.187	46.67	18	**0.0002**	11 (105)	**0.0203**	2	0.1973	-0.0356, 0.4302	0.0968

Diversity of fishery species	3	0.204	-0.023, 0.431	0.079	0.663	2	0.718	1 (25)	**0.0304**	2	0.1178	-0.0666, 0.3022	0.2105
Abundance of macrobenthos	405	0.058	-0.051, 0.166	0.296	21865.27	404	**0.0001**	6899 (2035)	**0.0001**	0			
Abundance of Annelida	204	-0.017	-0.175, 0.14	0.828	1802.915	203	**0.0001**	288 (1030)	**0.0054**	0			
Abundance of Arthropoda	67	0.233	-0.066, 0.532	0.127	574.886	66	**0.0001**	999 (345)	**0.0001**	1	0.2057	-0.0959, 0.5074	0.1814
Abundance of Nematoda	10	-0.645	-1.347, 0.056	0.071	34.047	9	**0.0001**	27 (60)	**0.0008**	1	-0.4499	-1.2137, 0.3139	0.2483
Biomass of macrobenthos	63	-0.106	-0.504, 0.292	0.601	375.548	62	**0.0001**	0 (325)	0.335	0			
Biomass of Annelida	9	0.319	-0.498, 1.136	0.443	25.238	8	**0.0014**	9 (55)	**0.0113**	1	0.4971	-0.3347, 1.3289	0.2415
Biomass of Arthropoda	22	0.377	-0.229, 0.982	0.223	63.345	21	**0.0001**	75 (120)	**0.0003**	0			
Biomass of Mollusca	7	-0.125	-0.916, 0.666	0.757	16.283	6	**0.012**	0 (45)	0.4275	3	0.6319	-0.308, 1.5718	0.1876
All variables in biodiversity conservation	506	0.088	-0.008, 0.185	0.0736	22595.85	505	**0.0001**	21799 (2540)	**0.0001**	78	0.3927	0.282, 0.504	**0.0001**
Size of fishery species	45	0.166	0.071, 0.26	**0.0006**	201.656	44	**0.0001**	586 (235)	**0.0001**	0			
Size of fishes	2	-0.058	-0.11, -0.005	**0.032**	0.0008	1	0.977	1 (20)	**0.0356**	0			
Size of Crustaceans	43	0.181	0.082, 0.279	**0.0003**	170.707	42	**0.0001**	669 (225)	**0.0001**	0			
Biomass of fishery species	28	1.757	1.102, 2.411	**0.0001**	202.06	27	**0.0001**	7117 (150)	**0.0001**	0			
Biomass of fishes	9	0.54	-0.965, 2.045	0.482	66.746	8	**0.0001**	15 (55)	**0.0042**	0			
Biomass of Crustaceans	13	2.37	1.493, 3.247	**0.0001**	87.783	12	**0.0001**	1455 (75)	**0.0001**	2	2.7536	1.823, 3.6843	**0.0001**
Abundance of fishery species	438	1.135	0.983, 1.286	**0.0001**	15777.745	437	**0.0001**	1405822 (2200)	**0.0001**	0			
Abundance of fishes	186	0.159	-0.0527, 0.3682	0.1417	987.7006	185	**0.0001**	1137 (940)	**0.0001**	30	-0.2221	-0.4571, 0.013	0.0641
Abundance of Crustaceans	193	1.98	1.787, 2.174	**0.0001**	1450.932	192	**0.0001**	230198 (975)	**0.0001**	30	1.6016	1.3796, 1.8236	**0.0001**
Abundance of Mollusca	52	0.4302	0.170, 0.691	**0.0012**	261.965	51	**0.0001**	1335 (270)	**0.0001**	0			
Catch rate of fishery species	68	0.598	0.367, 0.83	**0.0001**	307.107	67	**0.0001**	2451 (350)	**0.0001**	5	0.6892	0.4544, 0.9239	**0.0001**
Catch rate of fishes	26	0.488	0.091, 0.885	**0.016**	103.269	25	**0.0001**	164 (140)	**0.0001**	1	0.5176	0.1228, 0.9124	**0.0102**
Catch rate of Crustaceans	22	0.583	0.195, 0.972	**0.003**	26.85	21	0.176	73 (120)	**0.0003**	0			
All variables in fishery production	615	0.9556	0.838, 1.073	**0.0001**	22453.56	614	**0.0001**	1886434 (3085)	**0.0001**	56	1.194	1.067, 1.321	**0.0001**

Supplementary Table 9. Summary of random-effects model effect size estimates and publication bias tests for environment factors. Detailed test statistics are presented in Supplementary Table 5. Significant *P* values (< 0.05) are shown in bold. *P* values reflect two-sided tests.

Effect size metrics	Random-effects model				Homogeneity test			Fail-safe number test		Trimfill			
	n	estimate	95% CIs	*P*	Cochran's Q	df	*P*	FSN (5n + 10)	*P*	*n* added	estimate	95% CIs	*P*
**vs natural wetlands**													
Elevation	68	-0.5838	-0.9364, 0.2312	**0.0012**	348.7517	67	**0.0001**	1241 (350)	**0.0001**	0			
Bulk density	57	0.6542	0.4613, 0.8470	**0.0001**	8521.7815	56	**0.0001**	60531 (2950)	**0.0001**	0			
Gravel (grain size > 2mm)	15	0.887	0.0949, 1.6792	**0.0282**	132.5666	14	**0.0001**	193 (85)	**0.0001**	0			
Sand (63 um < grain size < 2 mm)	60	0.2094	0.014, 0.4049	**0.0357**	1314.977	59	**0.0001**	1130 (310)	**0.0001**	0			
Sand and gravel (grain size > 63 um)	6	0.312	0.066, 0.558	**0.0129**	113.5547	5	**0.0001**	139 (40)	**0.0001**	0			
Silt (2 um < grain size < 63 um)	26	-0.2449	-0.4817, -0.0081	**0.0426**	25835116	25	**0.0001**	166 (140)	**0.0001**	0			
Clay (grain size < 2 um)	50	-0.5602	-0.7026, -0.4177	**0.0001**	215.6538	49	**0.0001**	3428 (260)	**0.0001**	16	-0.3335	-0.4919, -0.1752	**0.0001**
Silt and clay (grain size < 63 um)	26	-0.4796	-0.8214, -0.1377	**0.006**	679.0515	25	**0.0001**	1798 (140)	**0.0001**	0			
Soil Moisture	19	-0.3424	-0.575, -0.1098	**0.0039**	1464.9959	18	**0.0001**	3094 (105)	**0.0001**	1	-0.3827	-0.6157, -0.1496	**0.0013**
Soil porewater salinity	22	0.206	0.1353, 0.2767	**0.0001**	178.8284	21	**0.0001**	4299 (120)	**0.0001**	7	0.2638	0.1914, 0.3362	**0.0001**
Soil porewater pH	48	0.0095	-0.0404, 0.0595	0.7081	1159.8815	47	**0.0001**	1652 (250)	**0.0001**	0			
Soil Redox potential (Eh)	53	0.9416	0.5623, 1.3208	**0.0001**	226.2652	52	**0.0001**	2588 (275)	**0.0001**	2	0.8741	0.48, 1.2681	**0.0001**
Surface water salinity	55	0.0094	-0.0389, 0.0576	0.7038	1168.0234	54	**0.0001**	4378 (285)	**0.0001**	0			
Surface water dissolved oxygen	44	0.0467	-0.0084, 0.1017	0.0967	265.7507	43	**0.0001**	0 (230)	0.0994	12	-0.0223	-0.0859, 0.0412	0.4909
Surface water temperature	49	0.0032	-0.0144, 0.0207	0.7251	450.9477	48	**0.0001**	0 (255)	0.2879	0			
Surface water turbidity	19	-0.0413	-0.2436, 0.1609	0.6889	48.8139	18	**0.0001**	0 (105)	0.1342	0			
**vs degraded wetlands**													
Elevation	77	-0.6586	-1.7865, 0.4692	0.2524	1054.2767	76	**0.0001**	337 (395)	**0.0001**	0			
Bulk density	53	0.0866	0.033, 0.1402	**0.0015**	138.2512	52	**0.0001**	435 (275)	**0.0001**	15	-0.0019	-0.0617, 0.0579	0.95
Sand (63 um < grain size < 2 mm)	11	0.4177	-0.3027, 1.1381	0.2557	610.8916	10	**0.0001**	367 (65)	**0.0001**	0			
Silt (2 um < grain size < 63 um)	6	-0.0144	-0.0877, 0.0589	0.6997	8.5991	5	0.1262	0 (40)	0.3224	3	-0.0551,	-0.1226, 0.0125	0.1102
Clay (grain size < 2 um)	6	0.0136	-0.0323, 0.0595	0.5601	7.1121	5	0.2124	0 (40)	0.2563	2	0.0224	-0.0186, 0.0633	0.285
Silt and clay (grain size < 63 um)	11	-0.132	-0.8965, 0.6325	0.735	108.5977	10	**0.0001**	3 (65)	**0.0346**	1	-0.3413	-1.1469, 0.4643	0.4064
Soil Moisture	10	-0.0602	-0.116, -0.0043	**0.0349**	7.8009	9	0.5543	16 (60)	**0.0044**	0			
Soil porewater salinity	13	0.0688	-0.0509, 0.1886	0.2597	182.7851	12	**0.0001**	28 (75)	**0.0018**	0			
Soil porewater pH	35	-0.0079	-0.017, 0.0012	0.0887	48.6154	34	**0.0499**	0 (185)	0.0756	7	-0.0149	-0.0259, -0.0039	**0.008**
Soil Redox potential (Eh)	44	0.5991	0.3872, 0.8109	**0.0001**	53.8089	43	0.1249	564 (230)	**0.0001**	0			
Surface water salinity	56	0.002	-0.0564, 0.0496	0.9347	1120.796	55	**0.0001**	164 (290)	**0.0006**	18	-0.0572	-0.1049, -0.0095	**0.0187**
Surface water dissolved oxygen	26	-0.0936	-0.1868, -0.0005	**0.0488**	982.8289	25	**0.0001**	858 (140)	**0.0001**	5	-0.154	-0.2449, -0.0631	**0.0009**
Surface water temperature	56	-0.0028	-0.0296, 0.024	0.838	981.1609	55	**0.0001**	909 (290)	**0.0001**	16	0.0321	0.0012, 0.063	**0.042**
Surface water turbidity	19	-0.1621	-0.3892, 0.065	0.1618	87.1945	18	**0.0001**	80 (105)	**0.0001**	0			

Supplementary Table 10. Summary of meta-regression models examining the effects of planted age on effects sizes. Shown are sample sizes (number of age gradients, number of observations), R^2, estimated intercept and correlation coefficient, and results of the moderator tests for the significance of the estimated correlation coefficient (Q_M and *P* values). *P* values reflect two-sided tests. Significant *P* values (< 0.05) are shown in bold. NA indicates no applicable due to low sample sizes.

Effect size metrics	vs natural wetlands						vs degraded wetlands					
	sizes	R^2 (%)	Intercept	Correlation coefficient	Q_M	*P*	sizes	R^2 (%)	Intercept	Correlation coefficient	Q_M	*P*
Vertical accretion rate	18, 43	< 0.01	0.195	-0.072	0.709	0.3996	18, 45	< 0.01	0.147	0.089	0.347	0.556
Elevation change rate	40, 75	< 0.01	0.528	0.055	0.194	0.66	15, 51	< 0.01	0.568	-0.09	0.442	0.506
Sediment deposition rate	8, 35	69.12	3.402	-1.561	43.77	**0.001**	3, 43	< 0.01	0.815	0.338	1.280	0.259
Shoreline change rate	10, 20	95.97	-0.009	0.014	1.640	0.2	12, 26	< 0.01	0.171	0.147	0.231	0.631
Plant density	32, 297	38.41	-1.268	0.813	142.8	**0.001**	6, 40	8.28	2.82	-0.913	3.363	0.067
Plant coverage	14, 109	21.7	-0.85	0.343	19.54	**0.001**	9, 52	< 0.01	1.924	-0.132	0.266	0.606
Plant height	15, 72	< 0.01	-0.149	0.069	1.411	0.235	3, 20	54.89	1.545	-1.158	7.716	**0.005**
Aboveground biomass	34, 288	1.78	-0.273	0.063	3.549	0.06	7, 59	31.83	2.032	-0.811	23.989	**0.001**
Belowground biomass	34, 244	35	-1.155	0.368	93.57	**0.001**	10, 45	3.44	1.853	-0.316	3.251	0.071
Net primary production	2, 27	NA	NA	NA	NA	NA	0, 0	NA	NA	NA	NA	NA
SOC content (top metre)	39, 219	< 0.01	-0.514	-0.049	0.644	0.422	13, 65	3.91	0.222	-0.108	2.141	0.143
SOC content (< 10 cm)	28, 128	1.68	-0.715	0.1	1.859	0.173	10, 36	10.12	0.357	-0.196	2.669	0.102
SOC content (0-20 cm)	12, 27	< 0.01	-0.558	0.058	0.102	0.75	1, 4	NA	NA	NA	NA	NA
SOC content (0-30 cm)	15, 29	< 0.01	-1.276	0.134	0.807	0.369	3, 9	69.4	0.223	-0.341	8.922	**0.003**
SOC content (>10 cm)	10, 14	12.23	-0.269	-0.43	1.912	0.167	0, 0	NA	NA	NA	NA	NA
SOC density (top metre)	14, 41	< 0.01	-0.61	-0.023	0.043	0.836	2, 18	NA	NA	NA	NA	NA
SOC density (<10 cm)	12, 13	57.85	-0.138	0.149	5.181	**0.023**	0, 0	NA	NA	NA	NA	NA
SOC density (0-20 cm)	0, 0	NA	NA	NA	NA	NA	0, 0	NA	NA	NA	NA	NA
SOC density (0-30 cm)	12, 14	< 0.01	-1.166	0.15	0.616	0.433	0, 0	NA	NA	NA	NA	NA
SOC density (>10 cm)	10, 14	< 0.01	-0.662	-0.13	0.385	0.535	0, 0	NA	NA	NA	NA	NA
SOC stocks	19, 79	20.07	-1.095	0.243	19.85	**0.001**	6, 9	83.76	0.195	0.335	28.056	**0.001**
SOC accumulation rate	2, 2	NA	NA	NA	NA	NA	0, 0	NA	NA	NA	NA	NA
CO_2 flux	5, 86	< 0.01	0.066	-0.03	0.04	0.841	0, 0	NA	NA	NA	NA	NA
CH_4 flux	3, 70	NA	NA	NA	NA	NA	0, 0	NA	NA	NA	NA	NA
N_2O flux	3, 70	NA	NA	NA	NA	NA	0, 0	NA	NA	NA	NA	NA
Species richness of vegetation	10, 25	10.3	-0.494	0.327	2.515	0.113	9, 19	43.62	1.326	-2.542	11.774	**0.001**
Species richness of birds	3, 5	53.75	3.747	-1.815	3.884	**0.049**	0, 0	NA	NA	NA	NA	NA
Species richness of macrobenthos	19, 59	31.81	-0.508	0.227	21.85	**0.001**	6, 20	5.73	0.007	0.155	1.567	0.211

Species richness of fishery species	9, 25	< 0.01	0.027	0.012	0.011	0.917	4, 13	3.66	0.961	-0.355	1.443	0.23
Species richness of fishes	1, 3	NA	NA	NA	NA	NA	1, 2	NA	NA	NA	NA	NA
Species richness of Crustaceans	1, 3	NA	NA	NA	NA	NA	1, 2	NA	NA	NA	NA	NA
Species richness of Mollusca	1, 3	NA	NA	NA	NA	NA	1, 2	NA	NA	NA	NA	NA
Diversity of vegetation	1, 2	NA	NA	NA	NA	NA	0, 0	NA	NA	NA	NA	NA
Diversity of birds	1, 2	NA	NA	NA	NA	NA	0, 0	NA	NA	NA	NA	NA
Diversity of macrobenthos	8, 21	1.29	-0.269	0.136	0.968	0.325	2, 15	NA	NA	NA	NA	NA
Diversity of fishery species	3, 3	100	-0.203	0.555	2.122	0.145	1, 3	NA	NA	NA	NA	NA
Evenness of vegetation	2, 6	NA	NA	NA	NA	NA	0, 0	NA	NA	NA	NA	NA
Evenness of birds	1, 2	NA	NA	NA	NA	NA	0, 0	NA	NA	NA	NA	NA
Evenness of macrobenthos	8, 13	43.25	0.264	-0.125	3.008	0.083	0, 0	NA	NA	NA	NA	NA
Abundance of birds	1, 18	NA	NA	NA	NA	NA	0, 0	NA	NA	NA	NA	NA
Abundance of macrobenthos	30, 334	5.91	-0.628	0.279	16.19	**0.001**	10, 239	4.54	-0.194	0.354	8.011	**0.005**
Abundance of Annelida	10, 105	1.84	-0.58	0.226	2.934	0.087	5, 83	5.53	-0.501	0.484	3.829	0.05
Abundance of Arthropoda	5, 64	< 0.01	0.004	-0.183	0.414	0.52	5, 34	5.33	-0.091	0.581	2.377	0.123
Abundance of Nematoda	3, 8	< 0.01	-0.781	1.22	0.570	0.45	4, 10	65.75	0.117	1.012	7.923	**0.005**
Biomass of macrobenthos	5, 62	< 0.01	-0.549	0.307	0.882	0.348	5, 63	30	-1.027	1.382	16.073	**0.001**
Biomass of Annelida	3, 12	< 0.01	-0.512	0.665	0.199	0.656	3, 9	< 0.01	-0.074	0.499	0.254	0.614
Biomass of Arthropoda	3, 25	2.88	0.023	-0.312	0.567	0.452	3, 22	< 0.01	0.502	-0.13	0.012	0.914
Biomass of Mollusca	3, 8	< 0.01	0.015	-1.538	1.308	0.253	3, 7	< 0.01	-0.408	0.382	0.147	0.701
Size of fishery species	5, 15	< 0.01	-0.053	-0.006	0.003	0.954	0, 0	NA	NA	NA	NA	NA
Size of fishes	0, 0	NA	NA	NA	NA	NA	0, 0	NA	NA	NA	NA	NA
Size of Crustaceans	5, 15	< 0.01	-0.053	-0.006	0.003	0.954	0, 0	NA	NA	NA	NA	NA
Biomass of fishery species	11, 64	< 0.01	0.127	-0.229	1.208	0.272	1, 28	NA	NA	NA	NA	NA
Biomass of fishes	6, 30	< 0.01	-0.294	-0.01	0.003	0.958	1, 9	NA	NA	NA	NA	NA
Biomass of Crustaceans	2, 24	NA	NA	NA	NA	NA	1, 13	NA	NA	NA	NA	NA
Abundance of fishery species	19, 517	< 0.01	-0.062	-0.002	0.001	0.971	5, 269	0.72	1.107	0.164	1.660	0.198
Abundance of fishes	19, 233	< 0.01	-0.115	0.074	0.634	0.426	5, 121	1.08	0.149	0.222	1.404	0.236
Abundance of Crustaceans	19, 219	< 0.01	-0.13	-0.042	0.245	0.62	5, 109	< 0.01	2.103	-0.02	0.016	0.898
Abundance of Mollusca	8, 60	5.29	-0.919	0.635	4.526	**0.033**	4, 43	< 0.01	0.186	0.243	0.699	0.403
Catch rate of fishery species	5, 33	37.02	0.091	-1.017	16.90	**0.001**	1, 24	NA	NA	NA	NA	NA
Catch rate of fishes	5, 15	44.25	0.372	-0.708	10.43	**0.001**	1, 8	NA	NA	NA	NA	NA
Catch rate of Crustaceans	2, 9	NA	NA	NA	NA	NA	1, 8	NA	NA	NA	NA	NA

Supplementary References

1. Anastasiou, C. J. & Brooks, J. R. Effects of soil pH, redox potential and elevation on survival of *Spartina patens* planted at a west central Florida salt marsh restoration site. *Wetlands* **23**, 845–859 (2003).
2. Angermeyer, A., Crosby, S. C., & Huber, J. A. Salt marsh sediment bacterial communities maintain original population structure after transplantation across a latitudinal gradient. *PeerJ*, **6**, e4735 (2018).
3. Armitage, A. R., Boyer, K. E., Vance, R. R. & Ambrose, R. F. Restoring assemblages of salt marsh halophytes in the presence of a rapidly colonizing dominant species. *Wetlands* **26**, 667-676 (2006).
4. Balouskus, R. G. & Targett, T. E. Egg deposition by Atlantic silverside, Menidia menidia: substrate utilization and comparison of natural and altered shoreline type. *Estuar. Coast.* **35**, 1100-1109 (2012).
5. Balouskus, R.G. & Targett, T.E. Fish and blue crab density along a Riprap-Sill-hardened shoreline: comparisons with Spartina Marsh and Riprap. *T. Am. Fish. Soc.* **145**, 766-773 (2016).
6. Beck, J. & Gustafson, D. J. Plant source influence on *Spartina alterniflora* survival and growth in restored South Carolina salt marshes. *Southeast. Nat.* **11**, 747-754 (2012).
7. Benner, C. S., Knutson, P. L., Brochu, R. A. & Hurme, A. K. Vegetative erosion control in an oligohaline environment Currituck Sound, North Carolina. *Wetlands* **2**, 105-117 (1982).
8. Bergen, A., Alderson, C., Bergfors, R., Aquila, C. & Matsil, M. A. Restoration of a *Spartina alterniflora* salt marsh following a fuel oil spill, New York City, NY. *Wetl. Ecol. Manag.* **8**, 185-195 (2000).
9. Boyer, K. E. & Zedler, J. B. Nitrogen addition could shift plant community composition

in a restored California salt marsh. *Restor. Ecol.* **7**, 74-85 (1999).

10. Broome, S. W. & Craft, C. B. Tidal salt marsh restoration, creation, and mitigation. *Reclamation of drastically disturbed lands. American Society of Agronomy, Madison, Wisconsin, USA*. **41**, 939-959 (2000).

11. Broome, S. W., Seneca, E. D. & Woodhouse, W. W. The effects of source, rate and placement of nitrogen and phosphorus fertilizers on growth of *Spartina alterniflora* transplants in North Carolina. *Estuaries* **6**, 212-226 (1983).

12. Broome, S. W., Seneca, E. D. & Woodhouse, W. W. Long-term growth and development of transplants of the salt marsh grass *Spartina alterniflora*. *Estuaries* **9**, 63-74 (1986).

13. Broome, S. W., Seneca, E. D. & Woodhouse, W. W. Tidal salt marsh restoration. *Aquat. Bot.* **32**, 1-22 (1988).

14. Cagle, G., Lin, Q., Graham, S. A., Mendelssohn, I., Fleeger, J. W., Deis, D., Johnson, D. S., Zhou, J. & Hou, A. Planting *Spartina alterniflora* in a salt marsh denuded of vegetation by an oil spill induces a rapid response in the soil microbial community. *Ecol. Eng.* **151**, 105815 (2020).

15. Cahoon, D. R., Lynch, J. C., Roman, C. T., Schmit, J. P. & Skidds, D. E. Evaluating the relationship among wetland vertical development, elevation capital, sea-level rise, and tidal marsh sustainability. *Estuar. Coast.* **42**, 1-15 (2019).

16. Calloway, J. C., Zedler, J. B. & Ross, D. L. Using tidal salt marsh mesocosms to aid wetland restoration. *Restor. Ecol.* **5**, 135-146 (1997).

17. Callaway, J. C., Sullivan, G. & Zedler, J. B. Species-rich plantings increase biomass and nitrogen accumulation in a wetland restoration experiment. *Ecol. Appl.* **13**, 1626-1639 (2003).

18. Castillo, J. M. & Figueroa, E. Restoring salt marshes using small cordgrass, *Spartina maritima*. *Restor. Ecol.* **17**, 324-326 (2009).

19. Chen, W., Ge, Z. M., Fei, B. L., Zhang, C., Liu, Q. X. & Zhang, Q. Soil carbon and nitrogen storage in recently restored and mature native *Scirpus* marshes in the Yangtze Estuary, China: Implications for restoration. *Ecol. Eng.* **104**, 150-157 (2017).

20. Chen X. et al. Restoring wetlands outside of the seawalls and to provide clean water habitat. *Sci. Total. Environ.* **721**, 137788 (2020).

21. Chen, Y. H., Yuan, L., Cao, H. B., Wang, H., Zhao, Z. Y., Niu, W. L. & Zhang, L. Q． The stress-gradient hypothesis and facilitation theory-based restoration technique for *Scirpus mariqueter* population． *Acta Ecologica Sinica* 3**9**, 4233-4241 (2019).

22. Chung, C. H., Zhuo, R. Z. & Xu, G. W. Creation of *Spartina* plantations for reclaiming Dongtai, China, tidal flats and offshore sands. *Ecol. Eng.* **23**, 135-150 (2004).

23. Clevering, O. A. & van Gulik, W. M. Restoration of *Scirpus lacustris* and *Scirpus maritimus* stands in a former tidal area. *Aquat. Bot.* **55**, 229-246 (1997).

24. Craft, C. B., Broome, S. W. & Seneca, E. D. Nitrogen, phosphorus and organic carbon pools in natural and transplanted marsh soils. *Estuaries* **11**, 272-280 (1988a).

25. Craft, C. B., Broome, S. W., Seneca, E. D. & Showers, W. J. Estimating sources of soil organic matter in natural and transplanted estuarine marshes using stable isotopes of carbon and nitrogen. *Estuar. Coast. Shelf. S.* **26**, 633-641 (1988b).

26. Craft, C. B., Broome, S. W. & Seneca, E. D. Exchange of nitrogen, phosphorus, and organic carbon between transplanted marshes and estuarine waters. *American Society of Agronomy, Crop Science Society of America, and Soil Science Society of America.* (1989).

27. Craft, C. B. Dynamics of nitrogen and phosphorus retention during wetland ecosystem succession. *Wetl. Ecol. Manag.* **4**, 177-187 (1997).

28. Crosby SC, Angermeyer A, Adler JM, Bertness MD, Deegan LA, Sibinga N, et al. *Spartina alterniflora* Biomass Allocation and Temperature: Implications for Salt Marsh Persistence with Sea-Level Rise. *Estuar. Coast.* **40**, 213-223 (2017).

29. Curado G, Figueroa E, Sanchez MI, Castillo JM. Avian communities in *Spartina maritima* restored and non-restored salt marshes. *Bird Study* **60**, 185-194 (2013a).

30. Curado G, Rubio-Casal AE, Figueroa E, Grewell BJ, Castillo JM. Native plant restoration combats environmental change: development of carbon and nitrogen sequestration capacity using small cordgrass in European salt marshes. *Environ. Monit. Assess.* **185**, 8439-8449 (2013b).

31. Curado G, Grewell BJ, Figueroa E, Castillo JM. Effectiveness of the Aquatic Halophyte *Sarcocornia perennis* spp. perennis as a Biotool for Ecological Restoration of Salt Marshes. *Water Air Soil Poll.* **225**, 1-14 (2014a).

32. Curado, G., Manzano-Arrondo, V., Figueroa, E., & Castillo, J. M. Public perceptions and uses of natural and restored salt marshes. *Landscape Research* **39**(6), 668-679 (2014b).

33. Curado G, Rubio-Casal AE, Figueroa E, Castillo JM. Plant Zonation in Restored, Nonrestored, and Preserved *Spartina maritima* Salt Marshes. *J. Coastal Res.* **30**, 629-634 (2014c).

34. Curado G, Rubio-Casal AE, Figueroa E, Castillo JM. Potential of *Spartina maritima* in Restored Salt Marshes for Phytoremediation of Metals in a Highly Polluted Estuary. *Int. J. Phytoremediat.* **16**, 1209-1220 (2014d).

35. Curado G, Gallego-Tevar B, Figueroa E, Castillo JM. Effects of removal of alien *Spartina densiflora* and restoration of native *S. maritima* on succession and zonation in European salt marshes. *Estuar. Coast. Shelf. S.* **244**, 105815 (2020).

36. Currin CA, Joye SB, Paerl HW. Diel rates of N-2-fixation and denitrification in a transplanted *Spartina alterniflora* marsh: Implications for N-flux dynamics. *Estuar. Coast. Shelf. S.* **42**, 597-616 (1996).

37. Currin CA, Delano PC, Valdes-Weaver LM. Utilization of a citizen monitoring protocol to assess the structure and function of natural and stabilized fringing salt marshes in North

Carolina. *Wetl. Ecol. Manag.* **16**, 97-118 (2008).

38. Currin CA, Chappell WS, Deaton A. Developing alternative shoreline armoring strategies: the living shoreline approach in North Carolina. *U.S. Geological Survey* (2010).

39. Currin, C. A., Davis, J., & Malhotra, A. Response of Salt Marshes to Wave energy provides Guidance for Successful Living Shoreline Implementation. *Living shorelines: The science and management of nature-based coastal protection* (2017).

40. Darnell, T. M., & Smith, E. H. Recommended design for more accurate duplication of natural conditions in salt marsh creation. *Environ. Manage.* **29**(6), 813-823 (2001).

41. Davenport TM, Seitz RD, Knick KE, Jackson N. Living Shorelines Support Nearshore Benthic Communities in Upper and Lower Chesapeake Bay. *Estuar. Coast.* **411**, S197-S206 (2018).

42. Donnelly M, Shaffer M, Connor S, Sacks P, Walters L. Using mangroves to stabilize coastal historic sites: deployment success versus natural recruitment. *Hydrobiologia* **803**, 389-401 (2017).

43. Emond, C., Lapointe, L., Hugron, S. & Rochefort, L. Reintroduction of salt marsh vegetation and phosphorus fertilisation improve plant colonisation on seawater-contaminated cutover bogs. *Mires Peat* **18**(17), 1-17 (2016).

44. Feher LC, Willis JM, Hester MW. Importance of Site History and Environmental Setting on Soil Properties in Restored Louisiana Back-Barrier Island Salt Marshes. *J. Coastal Res.* **34**, 58-66 (2018).

45. Foster-Martinez MR, Variano EA. Biosolids as a marsh restoration amendment. *Ecol. Eng.* **117**, 165-173 (2018).

46. Freitas RF, Schrack EC, He Q, Silliman BR, Furlong EB, Telles AC, et al. Consumer control of the establishment of marsh foundation plants in intertidal mudflats. *Mar. Ecol. Prog. Ser.* **547,** 79-89 (2016).

47. Gaynor ML, Walters LJ, Hoffman EA. Ensuring effective restoration efforts with salt marsh grass populations by assessing genetic diversity. *Restor. Ecol.* **27**, 1452-1462 (2019).

48. Gittman RK, Fodrie FJ, Baillie CJ, Brodeur MC, Currin CA, Keller DA, et al. Living on the Edge: Increasing Patch Size Enhances the Resilience and Community Development of a Restored Salt Marsh. *Estuar. Coast.* **41**, 884-895 (2018).

49. Handa, I. T. & Jefferies, R. L. Assisted revegetation trials in degraded salt-marshes. *J. Appl. Ecol.* **37**, 944-958 (2000).

50. Hu, Z. J. A study on population restoration techniques of *Scirpus mariqueter* in the newly formed tidal marsh. *East China Normal University*, 2017.

51. Hu, Z. J. et al. Revegetation of a native species in a newly formed tidal marsh under varying hydrological conditions and planting densities in the Yangtze Estuary. *Ecol. Eng.* **83**, 354–363 (2015).

52. Janousek, C. N., Currin, C. A. & Levin, L. A. Succession of microphytobenthos in a restored coastal wetland. *Estuar. Coast.* **30**, 265-276 (2007).

53. Johnson, D. S., Fleeger, J. W., Riggio, M. R., Mendelssohn, I. A., Lin, Q., Graham, S. A., Deis, D. R. & Hou, A. Saltmarsh plants, but not fertilizer, facilitate invertebrate recolonization after an oil spill. *Ecosphere* **9**, e02082 (2018).

54. Jones, S. F., Yando, E. S., Stagg, C. L., Hall, C. T. & Hester, M. W. Restoration Affects Sexual Reproductive Capacity in a Salt Marsh. *Estuar. Coast.* **42**, 976-986 (2019).

55. Kibler, K. M., Kitsikoudis, V., Donnelly, M., Spiering, D. W. & Walters, L. Flow-Vegetation Interaction in a Living Shoreline Restoration and Potential Effect to Mangrove Recruitment. *Sustainability* **11**, 3215 (2019).

56. Konisky, R. A. & Burdick, D. M. Effects of stressors on invasive and halophytic plants of New England salt marshes: A framework for predicting response to tidal restoration. *Wetlands* **24**, 434-447 (2004).

57. Lee, I., Park, S., Ryu, S. & Kobayashi, N. Ecological Restoration Index for Evaluation of Artificial Salt Marsh. *J. Coastal Res.* **27**, 959-965 (2011).

58. Manis, J. E., Garvis, S. K., Jachec, S. M. & Walters, L. J. Wave attenuation experiments over living shorelines over time: a wave tank study to assess recreational boating pressures. *J. Coast. Conserv.* **19**, 1-11 (2015).

59. Mavrodi, O. V., Jung, C. M., Eberly, J. O., Hendry, S. V., Namjilsuren, S., Biber, P. D., Indest, K. J. & Mavrodi, D. V. Rhizosphere microbial communities of *Spartina alterniflora* and *Juncus roemerianus* from restored and natural tidal marshes on Deer Island, Mississippi. *Front. Microbiol.* **9**, 1-13 (2018).

60. Maynard, C., McManus, J., Crawford, R. & Paterson, D. A comparison of short-term sediment deposition between natural and transplanted saltmarsh after saltmarsh restoration in the Eden Estuary (Scotland). *Plant Ecol. Divers.* **4**, 103-113 (2011).

61. Maynard, C. E. Saltmarshes on the fringe: restoring the degraded shoreline of the Eden estuary, Scotland. *University of St Andrews* 2014.

62. McClenachan, G. M., Donnelly, M. J., Shaffer, M. N., Sacks, P. E. & Walters, L. J. Does size matter? Quantifying the cumulative impact of small-scale living shoreline and oyster reef restoration projects on shoreline erosion. *Restor. Ecol.* **28**, 1365-1371 (2020).

63. Milano, G. R. Restoration of coastal wetlands in southeastern Florida. *Wetland J.* **11**, 15-24 (1999).

64. Montemayor, M. B., Price, J., Rochefort, L. & Boudreau, S. Temporal variations and spatial patterns in saline and waterlogged peat fields: 1. Survival and growth of salt marsh graminoids. *Environ. Exp. Bot.* **62**, 333-342 (2008).

65. Montemayor, M. B., Price, J., Rochefort, L. & Boudreau, S. Temporal variations and spatial patterns in saline and waterlogged peat fields: II. Ion accumulation in transplanted salt marsh graminoids. *Environ. Exp. Bot.* **69**, 87-94 (2010).

66. Montemayor, M. B., Price, J. & Rochefort, L. The importance of pH and sand substrate in the revegetation of saline non-waterlogged peat fields. *J. Environ. Manage.* **163**, 87-97 (2015).

67. Morgan, P. A. & Short, F. T. Using functional trajectories to track constructed salt marsh development in the Great Bay Estuary, Maine/New Hampshire, USA. *Restor. Ecol.* **10**, 461-473 (2002).

68. Muench, A. & Elsey-Quirk, T. Competitive reversal between plant species is driven by species-specific tolerance to flooding stress and nutrient acquisition during early marsh succession. *J. appl. Ecol.* **56**, 2236-2247 (2019).

69. Neff, K. P., Rusello, K. & Baldwin, A. H. Rapid Seed Bank Development in Restored Tidal Freshwater Wetlands. *Restor. Ecol.* **17**, 539-548 (2009).

70. O'Connor, M. I., Violin, C. R., Anton, A., Ladwig, L. M. & Piehler, M. F. Salt marsh stabilization affects algal primary producers at the marsh edge. *Wetl. Ecol. Manag.* **19**, 131-140 (2011).

71. Ogburn, M. B. & Alber, M. An investigation of salt marsh dieback in Georgia using field transplants. *Estuar. Coast.* **29**, 54-62 (2006).

72. Palinkas, C. M., Sanford, L. P. & Koch, E. W. Influence of Shoreline Stabilization Structures on the Nearshore Sedimentary Environment in Mesohaline Chesapeake Bay. *Estuar. Coast.* **41**, 952-965 (2018).

73. Piehler, M. F., Currin, C. A., Cassanova, R., Paerl, H. W. Development and N2-fixing activity of the benthic microbial community in transplanted *Spartina alterniflora* marshes in North Carolina. *Restor. Ecol.* **6**, 290-296 (1998).

74. Polk, M. A. & Eulie, D. O. Effectiveness of Living Shorelines as an Erosion Control Method in North Carolina. *Estuar. Coast.* **41**, 2212-2222 (2018).

75. Pomeroy, W. M., Gordon, D. K., & Levings, C. D. Experimental transplants of brackish

and salt marsh species on the Fraser River Estuary. *Department of Fisheries and Oceans, Resources Services Branch, West Vancouver Laboratory*. (1981).

76. Rozas, L. P., Caldwell, P. & Minello, T. J. The fishery value of salt marsh restoration projects. *J. Coastal Res.* **40**, 37-50 (2005).

77. Rozas, L. P. & Minello, T. J. Marsh terracing as a wetland restoration tool for creating fishery habitat. *Wetlands* **21**, 327-341 (2001).

78. Sharma, S., Goff, J, Cebrian, J. & Ferraro, C. A hybrid shoreline stabilization technique: Impact of modified intertidal reefs on marsh expansion and nekton habitat in the northern Gulf of Mexico. *Ecol. Eng.* **90**, 352-360 (2016).

79. Shiau, Y., Burchell, M. R. & Krauss, K. W. Plant biomass, carbon content, decomposition, and soil greenhouse gas fluxes to support carbon budget development for a created salt marsh in eastern North Carolina, USA, (2016).

80. Shiau, Y., Burchell, M. R., Krauss, K. W., Broome, S. W. & Birgand, F. Carbon storage potential in a recently created brackish marsh in eastern North Carolina, USA. *Ecol. Eng.* **127**, 579-588 (2019).

81. Short, F. T., Burdick, D. M., Short, C. A., Davis, R. C., & Morgan, P. A. Developing success criteria for restored eelgrass, salt marsh, and mudflat habitats. *Ecol. Eng.* **15**, 239-252 (2000).

82. Silinski, A., van Belzen, J., Fransen, E., Bouma, T. J., Troch, P., Meire, P., & Temmerman, S. Quantifying critical conditions for seaward expansion of tidal marshes: A transplantation experiment. *Estuar. Coast. Shelf. S.* **169**, 227-237 (2016).

83. SER [Society for Ecological Restoration]. USA: Texas: Lavaca Bay Restoration, Point Comfort, Calhoun County. https://www.ser-rrc.org/project/usa-texas-lavaca-bay-restoration-point-comfort-calhoun-county/ (2001).

84. SER [Society for Ecological Restoration]. Australia: Salt Marsh Restoration at the

Olympics 2000 Site (Homebush Bay). https://www.ser-rrc.org/project/australia-salt-marsh-restoration-at-the-olympics-2000-site-homebush-bay/ (2007a).

85. SER [Society for Ecological Restoration]. USA: California: Restoration of Batiquitos Lagoon (Carlsbad). https://www.ser-rrc.org/project/usa-california-restoration-of-batiquitos-lagoon-carlsbad/ (2007b).

86. SER [Society for Ecological Restoration]. USA: New York: Bar Beach Salt Marsh Restoration, Hempstead Harbor. https://www.ser-rrc.org/project/usa-new-york-bar-beach-salt-marsh-restoration-hempstead-harbor/ (2012).

87. SER [Society for Ecological Restoration]. USA: Maidford River Saltmarsh Restoration: Middletown, Rhode Island. https://www.ser-rrc.org/project/maidford-river-saltmarsh-restoration-middletown-rhode-island-usa/ (2015).

88. Sparks, E. L., Cebrian, J., Biber, P. D., Sheehan, K. L. & Tobias, C. R. Cost-effectiveness of two small-scale salt marsh restoration designs. *Ecol. Eng.* **53**, 250-256 (2013).

89. Sparks, E. L., Cebrian, J., Tobias, C. R., & May, C. A. Groundwater nitrogen processing in Northern Gulf of Mexico restored marshes. *J. Environ. Manage.* **150**, 206-215 (2015).

90. Spiering, D. W., Kibler, K. M. & Kitskoudis, V. Hydrodynamic change following living shoreline restoration based on a Before-After-Control-Impact experiment. *World Environmental and Water Resources Congress, American Society of Civil Engineers, Hydraulics and Waterways, Water Distribution Systems Analysis, and Smart Water*, 54-64 (2018).

91. Stalter, R. & Batson, W. T. Transplantation of salt marsh vegetation, Georgetown, South Carolina. *Ecology* **50**, 1087-1089 (1969).

92. Staszak, L. A. & Armitage, A. R. Evaluating Salt Marsh Restoration Success with an Index of Ecosystem Integrity. *J. Coast. Res.* **287**, 410–418 (2013).

93. Tao, Y. D., Zhong, S. C., Li, C. W., Zhao, M., Ding, P. Z., Fang, S. B., He, P. M., Yu, K. F.

A study on the effect of ecological restoration and reconstruction of *Scirpus mariqueter* community: A case of Nanhui coasts. *Transactions of Oceanology and Limnology* **5**, 40-49 (2018).

94. Taylor, B. W., Paterson, D. M. & Baxter, J. M. Sediment Dynamics of Natural and Restored *Bolboschoenus maritimus* Saltmarsh. *Front. Ecol. Evol.* **7**, 237 (2019).
95. Thompson, J. D., McNeilly, T. & Gray, A. J. Population variation in *Spartina anglica* C. E. Hubbard. II. Reciprocal transplants among three successional populations. *New Phytol.* 117, 129-139 (1991).
96. Thompson, S. P., Paerl, H. W. & Go, M. C. Seasonal patterns of nitrification and denitrification in a natural and a restored salt marsh. *Estuaries* **18**, 399-408 (1995).
97. Wilsey, B. J., McKee, K. L. & Mendelssohn, I. A. Effects of increased elevation and macro- and micronutrient additions on *Spartina alterniflora* transplant success in salt-marsh dieback areas in Louisiana. *Environ. Manage.* **16**, 505-511 (1992).
98. Xu, Y., Yao, S., Soetaert, K., Fan, X. Effects of salt marsh restoration on eukaryotic microbenthic communities in the Yangtze Estuary. *Mar. Ecol. Prog. Ser.* **638**, 39-50 (2020).
99. Yando, E. S., Osland, M. J., Jones, S. F., Hester, M. W. Jump-starting coastal wetland restoration: a comparison of marsh and mangrove foundation species. *Restor. Ecol.* **27**, 1145-1154 (2019).
100. Zedler, J. B. Canopy architecture of natural and planted cordgrass marshes: selecting habitat evaluation criteria. *Ecol. Appl.* **3**, 123–138 (1993).
101. Zedler, J. B., Callaway, J. C. & Sullivan, G. Declining biodiversity: Why species matter and how their functions might be restored in Californian tidal marshes. *BioScience* **51**, 1005-1017 (2001).
102. Zedler, J. B., Morzaria-Luna, H., Ward, K. The challenge of restoring vegetation on tidal, hypersaline substrates. *Plant Soil* **253**, 259-273 (2003).

103. Zedler, J.B. & West, J.M. Declining diversity in natural and restored salt marshes: a 30-year study of Tijuana Estuary. *Restor. Ecol.***16**, 249-262 (2008).

104. Zhou, C. F., Qin, P. & Xie, M. Vegetating coastal areas of east China: Species selection, seedling cloning and transplantation. *Ecol. Eng.* **20**, 275-286 (2003).
