# Supplementary material for: A global meta-analysis on the drivers of salt marsh planting success and implications for ecosystem services
Source: Nat Commun. 2024 Apr 29;15:3643. doi: 10.1038/s41467-024-47769-5 (PMC11059165; doi:10.1038/s41467-024-47769-5)
Supplement: Supplementary file 2 — Reporting Summary [file 41467_2024_47769_MOESM2_ESM.pdf]

nature portfolio

Corresponding author(s): Zhan Hu, Baoshan Cui

Last updated by author(s): Mar 27, 2024

Reporting Summary

Nature Portfolio wishes to improve the reproducibility of the work that we publish. This form provides structure for consistency and transparency in reporting. For further information on Nature Portfolio policies, see our Editorial Policies and the Editorial Policy Checklist.

Statistics

For all statistical analyses, confirm that the following items are present in the figure legend, table legend, main text, or Methods section.

n/a	Confirmed	
[ ]	[x]	The exact sample size (n) for each experimental group/condition, given as a discrete number and unit of measurement
[x]	[ ]	A statement on whether measurements were taken from distinct samples or whether the same sample was measured repeatedly
[ ]	[x]	The statistical test(s) used AND whether they are one- or two-sided<br>*Only common tests should be described solely by name; describe more complex techniques in the Methods section.*
[ ]	[x]	A description of all covariates tested
[ ]	[x]	A description of any assumptions or corrections, such as tests of normality and adjustment for multiple comparisons
[ ]	[x]	A full description of the statistical parameters including central tendency (e.g. means) or other basic estimates (e.g. regression coefficient) AND variation (e.g. standard deviation) or associated estimates of uncertainty (e.g. confidence intervals)
[x]	[ ]	For null hypothesis testing, the test statistic (e.g. F, t, r) with confidence intervals, effect sizes, degrees of freedom and P value noted<br>*Give P values as exact values whenever suitable.*
[x]	[ ]	For Bayesian analysis, information on the choice of priors and Markov chain Monte Carlo settings
[x]	[ ]	For hierarchical and complex designs, identification of the appropriate level for tests and full reporting of outcomes
[x]	[ ]	Estimates of effect sizes (e.g. Cohen's d, Pearson's r), indicating how they were calculated

Our web collection on statistics for biologists contains articles on many of the points above.

Software and code

Policy information about availability of computer code

Data collection	Quantitative data is extracted from the relevant peer-reviewed literature about the the drivers and impacts of salt marsh planting. The studies were identified through the Web of Science Core Collection. Latitude and longitude data were obtained by locating the study site on Google Earth, when the information is not available in the original text. Data from original figures were extracted using the online tool WebPlotDigitizer (https://apps.automeris.io/wpd/). Global coastline data is available at Natural Earth (naturalearthdata.com). Microsoft Excel 2019 was used for data collection.
Data analysis	All meta-analyses and meta-regressions were performed using the package 'metafor' in R software version 4.1.1. Global map was created using ArcGIS 10.2 software. Figures 1b,c, 2-5 were created using GraphPad Prism 8 (v8.00).

For manuscripts utilizing custom algorithms or software that are central to the research but not yet described in published literature, software must be made available to editors and reviewers. We strongly encourage code deposition in a community repository (e.g. GitHub). See the Nature Portfolio guidelines for submitting code & software for further information.

Data

Policy information about availability of data

All manuscripts must include a data availability statement. This statement should provide the following information, where applicable:

- Accession codes, unique identifiers, or web links for publicly available datasets
- A description of any restrictions on data availability
- For clinical datasets or third party data, please ensure that the statement adheres to our policy

All data supporting the findings of this study and used to produce the figures have been deposited to the online repository, Zenodo https://doi.org/10.5281/zenodo.10884395.

Research involving human participants, their data, or biological material

Policy information about studies with human participants or human data. See also policy information about sex, gender (identity/presentation), and sexual orientation and race, ethnicity and racism.

Reporting on sex and gender	Not relevant.
Reporting on race, ethnicity, or other socially relevant groupings	Not relevant.
Population characteristics	Not relevant.
Recruitment	Not relevant.
Ethics oversight	Not relevant.

Note that full information on the approval of the study protocol must also be provided in the manuscript.

Field-specific reporting

Please select the one below that is the best fit for your research. If you are not sure, read the appropriate sections before making your selection.

☐ Life sciences ☐ Behavioural & social sciences ☒ Ecological, evolutionary & environmental sciences

For a reference copy of the document with all sections, see nature.com/documents/nr-reporting-summary-flat.pdf

Ecological, evolutionary & environmental sciences study design

All studies must disclose on these points even when the disclosure is negative.

Study description	Here we present a global synthesis from 210 publications of planting outcomes in salt marshes. We quantified the survival and growth of outplants as success indicators and how there were affected by abiotic and biotic filters. We also examined the effects of salt marsh planting on a range of ecological benefits related to climate change mitigation and biodiversity conservation, and compared the outcomes to natural and degraded wetlands. For each variable related to shoreline protection, primary production, soil carbon storage, greenhouse gas (GHG) fluxes, biodiversity conservation and fishery production, we conducted a meta-analysis to quantify both log response ratios and Hedges' g* effect sizes, as a means of providing a quantitative estimate of salt marsh planting performance. Then, we examined how the effect sizes of planting varied with time since planting.
Research sample	We followed the PRISMA protocol for study selection and inclusion in the systematic review and meta-analysis. Finally, a total of 210 studies were retained. Overall 1,038 survival data from 45 plant species reported in 47 studies were found. 62 ecological benefits variables related to climate change mitigation and biodiversity conservation and 16 environmental variables were used in this study. To include the widest possible range of data, these studies that had at least one pair of data points of 78 variables comparing planted marshes versus adjacent natural or degraded wetlands were retained. Overall, 9,756 pairs of observations reported in 116 studies were retained. Of these, 6,377 pairs of observations from 102 publications were variables compared with natural wetlands, and 3,379 pairs of observations from 58 publications were variables compared with degraded wetlands. To estimate the effect sizes of planting, studies reporting mean values of the data with sample sizes and standard deviations/errors were retained. Overall, 6,570 pairs of observations reported in 115 studies were included. Of these, 4,449 pairs of observations from 101 publications compared variables with natural wetlands, and 2,121 pairs of observations from 57 publications compared variables with degraded wetlands. For each retained publication, we extracted the mean, statistical variation (i.e. standard error, standard deviation) and sample size for each variable at sites where planting was applied and natural or degraded sites from the main text, tables, or by digitizing figures in the articles. Finally, 1,038 survival data and 10,518 pairs of observations were included in this systematic review.
Sampling strategy	We followed the PRISMA protocol for study selection and inclusion. The peer-reviewed literature was searched through ISI Web of Science Core Collection. Details about the inclusion/exclusion criteria for publications are reported in Methods. We formalized the quality appraisal of the individual studies to assure the quality of studies included in the synthesis.
Data collection	ZZL collected/downloaded and preprocessed the data to a consistent form suitable for analysis. Details are reported in the Methods.

	We extracted mean, statistical variation (i.e. standard error, standard deviation) and sample size for restored and reference groups for each variable. When an original study reported the results graphically, we used webplotdigitizer to extract data from figures. We also extracted the information of study site (i.e. country, coordinates of study area), restoration method, restoration age, and restoration species origin for each case.
Timing and spatial scale	The analyzed studies were published between 1967 and 2020. Our study spans a total of 15 countries on five continents along the shorelines of the Atlantic, Pacific, and Indian Oceans. Most studies are in North America, Europe and East Asia. All data were collected in a search performed March 30, 2021
Data exclusions	No data were excluded.
Reproducibility	The methods of data collection and analysis are presented in the Methods section in detail and to enhance reproducibility the data supporting the results of this study are available from corresponding author.
Randomization	Randomization is not applicable to a meta-analysis.
Blinding	Blinding is not applicable for this study because there is no experiments carried out.

Did the study involve field work? ☐ Yes ☒ No

Reporting for specific materials, systems and methods

We require information from authors about some types of materials, experimental systems and methods used in many studies. Here, indicate whether each material, system or method listed is relevant to your study. If you are not sure if a list item applies to your research, read the appropriate section before selecting a response.

Materials & experimental systems

n/a	Involved in the study
☒	☐ Antibodies
☒	☐ Eukaryotic cell lines
☒	☐ Palaeontology and archaeology
☒	☐ Animals and other organisms
☒	☐ Clinical data
☒	☐ Dual use research of concern
☒	☐ Plants

Methods

n/a	Involved in the study
☒	☐ ChIP-seq
☒	☐ Flow cytometry
☒	☐ MRI-based neuroimaging

Plants

Seed stocks	Not relevant.
Novel plant genotypes	Not relevant.
Authentication	Not relevant.
