# Supplementary material for: A global meta-analysis on the drivers of salt marsh planting success and implications for ecosystem services
Source: Nat Commun. 2024 Apr 29;15:3643. doi: 10.1038/s41467-024-47769-5 (PMC11059165; doi:10.1038/s41467-024-47769-5)
Supplement: Supplementary file 3 — Description of Additional Supplementary Files [file 41467_2024_47769_MOESM3_ESM.pdf]

Description of Additional Supplementary Files

File name: Supplementary data 1

Description: Summary of ecological outcomes of planting in salt marshes.

File name: Supplementary data 2

Description: List of publications used in this systematic review.
